# SUMO regulates p21^Cip1^ intracellular distribution and with p21^Cip1^ facilitates multiprotein complex formation in the nucleolus upon DNA damage

**DOI:** 10.1371/journal.pone.0178925

**Published:** 2017-06-05

**Authors:** Sonia Brun, Neus Abella, Maria T. Berciano, Olga Tapia, Montserrat Jaumot, Raimundo Freire, Miguel Lafarga, Neus Agell

**Affiliations:** 1 Departament Biomedicina, Universitat de Barcelona, IDIBAPS, Barcelona, Spain; 2 Departamento de Anatomía y Biología Celular, Universidad de Cantabria-IDIVAL, Santander, Spain; 3 Unidad de Investigación, Hospital Universitario de Canarias, Instituto de Tecnologías Biomédicas, Tenerife, Spain; German Cancer Research Center, GERMANY

## Abstract

We previously showed that p21^Cip1^ transits through the nucleolus on its way from the nucleus to the cytoplasm and that DNA damage inhibits this transit and induces the formation of p21^Cip1^-containing intranucleolar bodies (INoBs). Here, we demonstrate that these INoBs also contain SUMO-1 and UBC9, the E2 SUMO-conjugating enzyme. Furthermore, whereas wild type SUMO-1 localized in INoBs, a SUMO-1 mutant, which is unable to conjugate with proteins, does not, suggesting the presence of SUMOylated proteins at INoBs. Moreover, depletion of the SUMO-conjugating enzyme UBC9 or the sumo hydrolase SENP2 changed p21^Cip1^ intracellular distribution. In addition to SUMO-1 and p21^Cip1^, cell cycle regulators and DNA damage checkpoint proteins, including Cdk2, Cyclin E, PCNA, p53 and Mdm2, and PML were also detected in INoBs. Importantly, depletion of UBC9 or p21Cip1 impacted INoB biogenesis and the nucleolar accumulation of the cell cycle regulators and DNA damage checkpoint proteins following DNA damage. The impact of p21Cip1 and SUMO-1 on the accumulation of proteins in INoBs extends also to CRM1, a nuclear exportin that is also important for protein translocation from the cytoplasm to the nucleolus. Thus, SUMO and p21^Cip1^ regulate the transit of proteins through the nucleolus, and that disruption of nucleolar export by DNA damage induces SUMO and p21^Cip1^ to act as hub proteins to form a multiprotein complex in the nucleolus.

## Introduction

The nucleolus is a non-membrane bound subnuclear organelle in which rRNAs are transcribed, processed, and assembled with ribosomal proteins into mature ribosomes [1,2]. The nucleolus contains three distinct compartments: the fibrillar center (FC), the dense fibrillar component (DFC), and the granular component (GC). Pre-rRNA is transcribed from rDNA at the border between the FC and the DFC. The FC is enriched in components of the RNA polymerase I transcription machinery, such as UBF, whereas the DFC contains various RNA modifying enzymes and pre-rRNA processing factors such as fibrillarin and small nucleolar ribonucleoproteins (snoRNPs). The GC is where pre-ribosome assembly takes place and is enriched in nuclephosmin/B23 [2,3]. Recent studies have suggested that in addition to ribosome biogenesis, the nucleolus is also associated with biological functions including cell cycle regulation, stress responses, heterochromatin maintenance, and capture and immobilization of proteins containing nucleolar detention sequences (NoDS) [2,4–7]. The maintenance of nucleolar organization relies on the equilibrium between transcription and processing of pre-rRNAs, and the export of the ribosomal subunits, with disruption of any of these processes ultimately affecting nucleolar structural integrity. Thus, a nucleolar-dependent response is observed after stress, such as radiation, or exposure to cytotoxic, proteotoxic or genotoxic agents [4,8,9], which directly or indirectly affect the structural integrity of the nucleolus. For example, functional nucleoli were proposed to be required for maintaining low levels of p53, which rise rapidly in response to stress-induced nucleolar impairment, leading to cell cycle arrest or apoptosis [10]. Several mechanisms underlie the increase in p53 upon nucleolar disruption, such as the release of the nucleolar proteins nucleophosmin or ARF into the nucleoplasm or the redirection of the 5S rRNA/ RPL11/ RPL5 pre-ribosomal complex, to the binding and inhibition of Mdm2, leading to p53 stabilization [4,11–15].

During ribosome biogenesis, equimolar amounts of ribosomal proteins are translated in the cytoplasm and imported into the nucleus. Then, the 40S and 60S subunits are exported from the GC to the cytoplasm by the adaptor protein NMD3 and the export factor CRM1 through the nuclear pores [16,17]. These import and export routes are prominent in cancer cells, which exhibit high rates of ribosome biogenesis and protein translation [18–21]. Indeed, enlarged nucleoli and increased contact between nucleoli and the nuclear envelope are observed in cancer cells [22] and is proposed that some proteins take advantage of this active route to translocate to the cytoplasm from the nucleus. Thus, there is a new emerging function of the nucleolus in regulating the nuclear-cytoplasmic transit of specific proteins, including those involved with cellular stress responses, such as p53 [10,23,24] and its downstream targets MDM2 and p21^Cip1^ (p21) [25]. Interestingly, p21 has a dual function in oncogenesis depending on its intracellular localization. It is oncogenic in the cytoplasm, but has a role as tumour suppressor in the nucleus, and therefore its intracellular traffic is highly regulated [26–31]. We previously demonstrated in normally growing cells that p21 transited through the nucleolus, but that DNA damage-induced nucleolar disruption inhibited this nuclear export and caused nuclear and nucleolar accumulation of p21. Under stress conditions, we detected p21 within the nucleolus in spherical aggregates that could be observed with different refringence under phase-contrast microscopy. We referred to these structures as intranucleolar bodies (INoBs), which could also be observed by electron microscopy within the coalescing non-electron dense regions of the GC [25]. The DNA damage checkpoint protein Rad9B is also recruited to this compartment upon nucleolar stress [32]. Interestingly, others have reported the presence of an intranucleolar body in normally growing cells that contained proteins involved in DNA maintenance, protein turnover, RNA metabolism, chromatin organisation and the small ubiquitin-like modifier (SUMO) [33].

The small ubiquitin-like modifier (SUMO) plays an important role in preserving the integrity and function of subnuclear compartments, including the nucleolus and promyelocytic leukaemia (PML) bodies, and also regulating communication between the nucleus and the cytoplasm [34–40]. Moreover, SUMOylation is widely involved in the DNA damage response [41,42]. The three different SUMO isoforms (SUMO-1-3) can be found free or covalently bound to proteins whose activity, interactions, and intracellular localization they regulate. Similar to ubiquitination, SUMOylation is an evolutionarily conserved three-step process involving an E1 activating enzyme, a single E2 conjugating enzyme (UBC9), which directly interacts with the substrate, and the substrate-specific E3 ligases [43,44]. Deconjugation of SUMO from target proteins involves SUMO-specific proteases (SENPs), of which six members have been identified, SENP1, 2, 3, 5, 6, and 7 [45]. Of these, SENP1 and SENP2 have broad specificity for the three mammalian SUMOs, while the others favour SUMO-2/3 as the substrate. SENP1, 6, and 7 localize in the nucleoplasm, but not in the nucleolus, SENP2 is nuclear and associates with the nuclear pores and nuclear speckles, while SENP3 and SENP5 are located in the nucleolus and are important for ribosome maturation and nucleolar export [46,47]. Thus, it has been proposed that SUMO-dependent subnuclear trafficking may assist in coordinating the rate of ribosome biogenesis [36]. Notably, mouse cells deficient in the UBC9 protein showed striking morphological nucleolar disruption [37]. Furthermore, SUMOylation of pre-ribosomal particles in the nucleus and subsequent deSUMOylation at the nuclear pore complex are necessary for efficient ribosome biogenesis and export in yeast [48].

Based on our previous studies on p21 and given that SUMO has been linked to nuclear and nucleolar protein export and multiprotein complex formation, we aimed to determine whether SUMOylation/deSUMOylation is involved in the nuclear/cytoplasmic distribution of p21, and investigated the role of p21 and SUMOylation in INoB generation upon DNA damage. In fact, we demonstrate that INoB biogenesis, and the concomitant accumulation in the INoB of proteins related to cell cycle regulation, DNA maintenance and chromatin organization is dependent on p21 and UBC9.

## Materials and methods

### Cell culture, RNA interference (siRNA), and gene transfer

HCT116 (ATCC collection) and HCT116 p21KO [49] cells were grown in DMEM/Ham’s F-12 (1:1) media supplemented with 10% fetal calf serum (FCS). DNA damage was induced by incubating with Adriamycin^®^ (Sigma) at a final concentration of 0.15 μg/ml for 24 hours, unless otherwise indicated.

To knock down proteins using siRNA, 2.4 x 10^5^ HCT116 cells in P35 dishes were transfected with the siRNA of interest at 50 nM, using HiPerFect Transfection Reagent (Qiagen) according to the manufacturer’s guidelines. After transfection, the cells were cultured for 48 hours.

DNA and shRNA expression in mammalian cells was achieved by transfecting cells with the appropriate expression vector and using Lipofectamine 2000 Reagent (Invitrogen), according to the manufacturer’s instructions. After transfection, the cells were cultured for 48 hours.

### siRNAs and cDNA constructs

The following commercial siRNAs were used: non-targeting siRNA (ON-TARGETplus^™^ Non-Targeting Control siRNA/ REF. D-001810-10-05/ Dharmacon Thermo Fisher); p21 siRNA (ON-TARGETplus^™^ SMARTpool siRNA/ REF. J-003471-09-0005 CDKN1A/ Dharmacon Thermo Fisher); UBC9 siRNA (ON-TARGETplus^™^ Human UBE2I(7329) SMARTpool siRNA/ REF. L-004910-00-0005/ Dharmacon Thermo Fisher); and SENP2 siRNA (ON-TARGETplus^™^ Human SENp2(59343) SMARTpool siRNA/ REF. L-006033-00-0005/ Dharmacon Thermo Fisher).

The following cDNA constructs were used: wild-type pEGFP-p21 and pMT2HA-p21 [27,28]; pEGFP-SUMO-1 (gift from Hay RT, University of Dundee, UK [50]); GFP-SUMO-1ΔGly-GLy, obtained by PCR using the appropriate primers to introduce a stop codon before the last two glycines in SUMO-1; Orange-SUMO-1, obtained by substituting Orange for GFP in the GFP-SUMO-1 plasmid using AgeI/BsrgI restriction enzymes; pECFP-CDK2; and pSUPER-puro-EGFP-p21, used to knock down p21 expression [51].

### Western blot analysis and antibodies

Cells were lysed in a buffer containing 2% SDS and 67 mM Tris-HCl, pH 6.8, before being sonicated for 20 seconds twice. The same amount (30 μg) of protein (measured by the Lowry method) from each cell lysate were resolved by SDS-polyacrylamide gel electrophoresis (SDS-PAGE) and transferred on to Immobilon-P membranes for 2 hours at 60 V. The membranes were incubated with the following antibodies: anti-p21Waf-1 mouse monoclonal antibody (diluted 1:200; Ab-1, OP64, Calbiochem); anti-UBC9 (UBE2I) rabbit polyclonal antibody (diluted 1:300; ab-30505, Abcam); anti-SENP2 rabbit polyclonal antibody (diluted 1:3,000; made by immunizing a rabbit with the antigen containing the amimoacids 111–358 of human SENP2); anti-SUMO-1 (FL-101) rabbit polyclonal antibody (sc-9060, Santa Cruz Biotechnology); and anti-actin (Clone C4) mouse monoclonal antibody (diluted 1:5,000; 691001, MP Biomedicals). After incubation with the appropriate peroxidase-coupled secondary antibody (diluted 1:2,500; Bio-Rad), immunocomplexes were detected by enhanced chemiluminescence (ECL) (Biological Industries).

### Immunofluorescence microscopy

HCT116 cells were grown on coverslips and treated as indicated above. For detection of GFP-fused proteins and/or Orange-fused proteins, HCT116 cells were fixed for 15 minutes in 4% paraformaldehyde, washed three times with phosphate-buffered saline (PBS), and mounted on glass slides with Mowiol (Calbiochem). For most endogenous protein or ectopic HA-p21 immunostaining, cells were fixed as above and permeabilized with 0.2% Triton X-100 in PBS for 10 minutes. For Cdk2 and PCNA immunodetection, cells were fixed with 4% paraformaldehyde for 1 hour. Cdk2 permeabilization was achieved with 0.2% Triton X-100 for 20 minutes and PCNA permeabilization was performed with methanol (100% at -20°C) for 4 minutes. Blocking was performed using 1% bovine serum albumin (BSA) in PBS. Cells were incubated with the primary antibodies overnight at 4°C, with the antibodies diluted in PBS containing 1% BSA. The antibodies used were the following: anti-p21Waf-1 mouse monoclonal antibody (diluted 1:200; Ab-1, OP64, Calbiochem); anti-p21 rabbit polyclonal antibody (diluted 1:200; C-19, sc-397, Santa Cruz Biotechnology); anti-UBF mouse monoclonal antibody (diluted 1:100; F-9, sc-13125, Santa Cruz Biotechnology); anti-fibrillarin rabbit polyclonal antibody (diluted 1:50; H-140, sc-25397, Santa Cruz Biotechnology); anti-p53 mouse monoclonal antibody (diluted 1:25; Pab 240, sc-99, Santa Cruz Biotechnology); anti-Mdm2 mouse monoclonal antibody (diluted 1:100; Ab-1, OP46, Calbiochem); anti-Cyclin E mouse monoclonal antibody (diluted 1:50; HE-12, sc-247, Santa Cruz Biotechnology); anti-SUMO-1 mouse monoclonal antibody (anti-GMP-1, diluted 1:100; 33–2400, Zymed), anti-SUMO-1 rabbit polyclonal antibody (FL-101, sc-9060, Santa Cruz Biotechnology); anti-UBC9 rabbit polyclonal antibody (diluted 1:50; ab30502, Abcam); anti-UBC9 rabbit monoclonal (diluted 1:50; D26F2 Cell Signaling); anti-HA mouse monoclonal antibody (diluted 1:200; 12CA5, 11 583816 001, Roche); anti-PML rabbit polyclonal antibody (diluted 1:200; H-238, sc-5621, Santa Cruz Biotechnology); anti-CRM1 (exportin-1) mouse monoclonal antibody (diluted 1:100; 611832, BD Biosciences); anti-Cdk2 rabbit polyclonal antibody (diluted 1:50; sc-163, Santa Cruz Biotechnology); and anti-PCNA mouse monoclonal antibody (diluted 1:50; Ab-1, NA03, Oncogene). Incubation with the secondary antibodies was performed following standard protocols. Coverslips were mounted on glass slides with Mowiol (Calbiochem).

To detect newly synthesized RNA, 5’-fluorouridine (5’-FU; Sigma-Aldrich, UK) was added to the culture medium at 2 mM for 20 min. The cells were then fixed in 3.7% paraformaldehyde in HPEM buffer (HPEM 2x: 60 mM Hepes, 130 mM Pipes, 20 mM EGTA, and 4 mM MgCl2.6H2O) containing 0.5% Triton X-100 for 10 min. The incorporation of the 5’-FU into nascent RNA was detected by incubation for 1 h at 37°C with a mouse monoclonal anti-BrdU antibody (clone BU-33, Sigma-Aldrich, UK), diluted 1:50 in PBS. The samples were then washed in 0.01% Tween 20 in PBS, incubated for 45 min with anti-mouse FITC-conjugated secondary antibody (Jackson ImmunoResearch), and mounted with the anti-fading medium Vectashield (Vector Laboratories).

Images were acquired using a Leica TCS SL laser scanning confocal spectral microscope (Leica Microsystems Heidelberg GmbH, Manheim, Germany), equipped with Argon and HeNe lasers and an inverted microscope. Alexa Fluor 488 (Molecular Probes), GFP and Alexa Fluor 594 (Molecular Probes), Orange and Alexa Fluor 647 (Molecular Probes), and phase-contrast images were acquired sequentially with the HCX PL APO CS 63.0 x 1.32 oil PH immersion objective (NA 1.32) and the excitation beam splitter 488/543/633 nm, using 488-, 543-, and 633-nm laser lines, and detection ranges of 500–540 nm, 585–700 nm, and 750–900 nm, respectively (pinhole of 1 airy unit).

INoB size was quantified using the Image J program. The set scale of the image was determined and then the length of the INoB measured. Statistical analysis (the t-test) was performed using the GraphPad Prism 6 software. Values significantly different using unpaired t-test were marked with asterisks are ** p < 0.01, *** p < 0.001, **** p < 0.0001).

### Electron microscopy

For immunogold electron microscopy, HCT116 cells were fixed with 4% paraformaldehyde in 0.1 M cacodylate buffer for 30 minutes at room temperature. Cells were scraped from the dishes, transferred to an Eppendorf tube, and centrifuged for 1 minute in a microfuge to obtain cell pellets. The pellets were washed with 0.1 M cacodylate buffer, dehydrated in increasing concentrations of methanol at -20°C, embedded in Lowicryl K4M at -20°C, and polymerized with ultraviolet irradiation. Ultrathin sections were mounted on nickel grids and sequentially incubated with 0.1 M glycine in PBS for 15 minutes, 5% BSA in PBS for 1 hour, and the rabbit polyclonal anti-SUMO-1 antibody (FL-101, sc-9060, Santa Cruz Biotechnology) diluted 1:50 in PBS containing 1% BSA and 0.1 M glycine for 1 hour. After washing, the sections were incubated with goat anti-rabbit IgG coupled to 10-nm gold particles (diluted 1:50 in PBS containing 1% BSA; BioCell, UK). After immunogold labeling, the grids were stained with lead citrate and uranyl acetate and examined with a Philips EM208 electron microscope operated at 60 kV. For control, ultrathin sections were treated as described above, but without the primary antibodies.

### Autoradiographic analysis of rRNA processing

RNA processing was analyzed as described in [14]. Briefly, 2.4 x 10^5^ cells/well in 6-well plates were transfected with the appropriate siRNA for 48 hours. Newly synthesized RNA was then labeled by incubating for 1 hour in a medium containing 1.2 μCi [5.6-^3^H]-uridine (Perkin Elmer) per ml of medium. Pulse-labeled cells were then washed with media containing 1 mM non-radioactive uridine (Sigma) and incubated for 4 hours at 37°C in 5% CO_2_. Following extraction, 1 μg of total RNA was size-separated by electrophoresis on a 1% agarose-formaldehyde gel.

### Measurement of new protein synthesis by ^3^H-leucine incorporation

To label newly synthesized proteins, 2.4 x 10^5^ cells/well in 12-well plates were incubated for 30 minutes at 37°C with 10 μCi ^3^H-Leu/ml of medium. Cells were washed with cold PBS, lysed with RIPA buffer, and then centrifuged to obtain pellets. The supernatant was precipitated with 20% cold trichloroacetic acid (TCA) for 10 minutes at 4°C. After centrifugation, the pellet was washed twice with 5% TCA and resuspended in 0.1 M NaOH. Of this, 70% was used to count the cpm in a liquid scintillation counter and the remainder used to determine protein concentration using the BCA protein assay. The counted cpm was divided by the protein concentration measured. Cells pre-treated with 100 μg/ml of cycloheximide were used as a positive control for protein synthesis inhibition. Assays were performed in triplicates.

## Results

### SUMO-1 and p21 colocalize in INoBs after DNA damage

**Fig 1:**
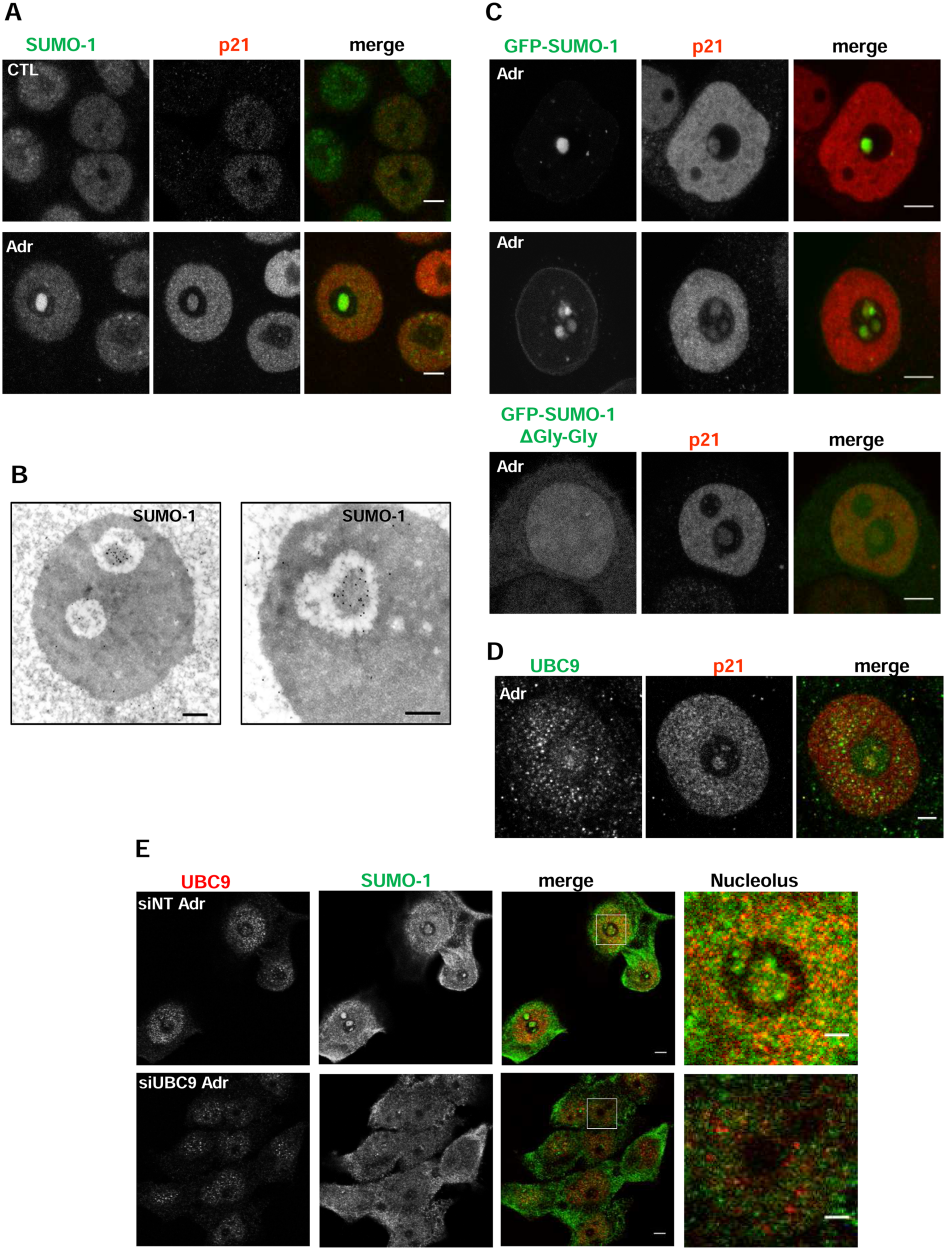
p21 and SUMO-1 colocalize in the disrupted nucleolus upon DNA damage. A) Immunodetection of endogenous SUMO-1 (green) using anti-SUMO-1 mouse antibody and p21 (red) using anti-p21 rabbit antibody in control (CTL) or treated with Adr for 48 hours (Adr) HCT116 cells. Scale bar: 5μm. B) Immunogold electronic microscopy of SUMO-1 showing the presence of SUMO-1 in the INoB HCT116 cells treated with Adr for 24 hours. Scale Bar: 0.5μm. C) Immunostaining of endogenous p21 (red) in GFP-SUMO-1 (two representative cells are shown) or GFP-SUMO-1ΔGly-Gly transfected cells treated 24h with Adr. Scale bar: 5μm. D) Immunostaining of p21 (red) and UBC9 (green) (rabbit polyclonal antibody) in 24-h Adr-treated HCT116 cells. Scale bar: 5μm. E) Immunostaining of SUMO-1 (green) and UBC9 (red) (rabbit monoclonal antibody) of 24-h Adr-treated HCT116 cells transfected with non-targeting (siNT) or UBC9 (siUBC9). Scale bar: 5 μm.

We previously demonstrated that Adriamycin (Adr)-induced DNA damage or other types of nucleolar stress disrupted the nucleolus and induced the formation of an INoB containing p21 [25]. This structure is dynamic and present in approximately 30% of the HCT116 cells treated with Adr for 24 hours. Interestingly, as shown in S1 Fig, this structure clearly correlates with a strong reduction of RNA transcription in the nucleoli even after recovery from Adr treatment. As protein SUMOylation is important for nuclear and nucleolar architecture and for multiprotein complex formation [35,37,40,52], we determined whether SUMO-1 colocalized with p21 in the nucleolus following Adr-induced DNA damage. Immunocytochemical analysis of endogenous SUMO-1 localization revealed mainly nucleoplasmic staining in non-treated cells; however, after Adr treatment, a strong nucleolar signal for SUMO-1 was observed colocalizing p21, indicating its localization in INoBs (Fig 1A). Similar results were obtained using a different anti-SUMO-1 antibody (S2A Fig). Immunogold electron microscopic analysis confirmed the preferential distribution of SUMO-1 immunoreactivity in the INoBs formed after the induction of DNA damage, which we have previously observed to contain p21 [25] (Fig 1B). These findings were confirmed by ectopic expression of a GFP-SUMO-1, which colocalized with p21 in the INoBs after Adr treatment, and was also found in the nuclear envelope (Fig 1C). Nucleolar localization was not observed with GFP-SUMO-1ΔGly-Gly, a SUMO-1 mutant that cannot be conjugated to proteins, indicating that wild type SUMO-1 is conjugated to proteins in p21-positive INoBs after DNA damage. Consistent with these observations we found that UBC9, the only known SUMO E2 enzyme, was also present in the INoB following DNA damage (Fig 1D and 1E). Most interestingly, depletion of UBC9 caused a decrease in SUMO-1 in the INoBs (Fig 1E and S2B Fig). All this suggests that protein SUMOylation actively occurs in these nucleolar structures.

### UBC9 and SENP2 regulate the nuclear/cytoplasmic transport of p21

**Fig 2:**
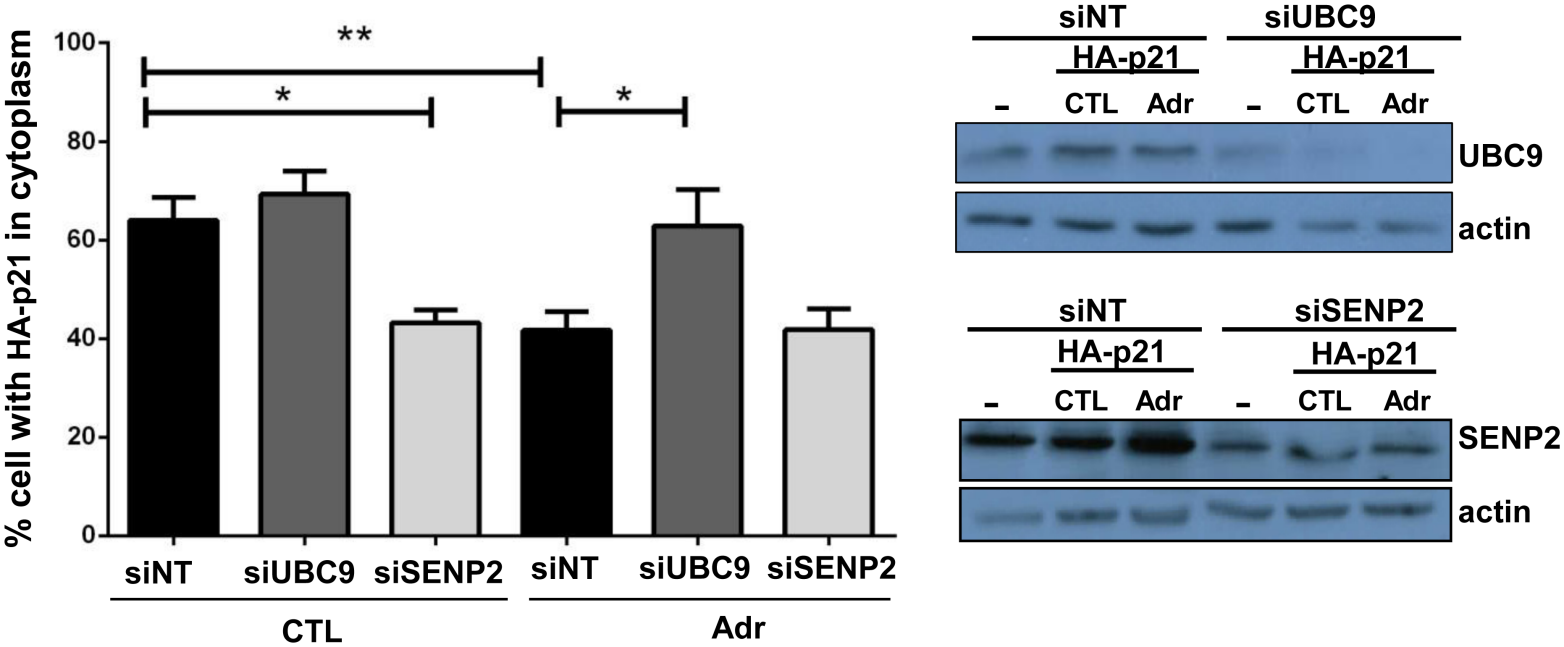
p21 nuclear/cytoplasmic transport of p21 is regulated by SENP2 and UBC9. Left: Graph shows the percentage of HCT116 cells with HA-p21 present in the cytoplasm determined by immunofluorescence analysis using anti-HA (Roche). Representative images are shown in S3A Fig. 48 hours after transfection with non-targeting (siNT), UBC9 (siUBC9), or SENP2 (siSENP2) siRNAs, cells were transfected with HA-p21 plasmid and 24 hours after treated (Adr) or not (Ctrl) with Adr for 12 hours. Data are the average of 3 different experiments and in each one at least 200 HA-p21 transfected cells were counted per condition. Rigth: Western Blotting to detect UBC9 or SENP2 levels in non-depleted or depleted-HCT116 cells; (-): correspond to cells after 48h of transfection with siRNA and priori to HA-p21 transfection.

As we previously demonstrated that INoB formation upon DNA damage not only correlated with p21 accumulation in the nucleolus, but also with the inhibition of the nuclear export of p21 to the cytoplasm we determined whether protein SUMOylation/deSUMOylation cycle regulated the nuclear-cytoplasmic distribution of p21. To test this possibility, we depleted UBC9 or SENP2 (the only know SUMO-1 hydrolase with nuclear and nuclear pore localization [45]). As we previously demonstrated, when HCT116 cells were transfected with HA-p21, the percentage of cells with cytoplasmic p21 significantly decreased upon Adr treatment as compared to non-treated cells (Fig 2 and S3A Fig). Importantly, this decrease was abolished in cells depleted of UBC9, indicating that a lack of SUMOylation impairs the nuclear accumulation of p21 induced by DNA damage. That this effect is dependent on DNA damage is shown by the fact that the depletion of UBC9 has no effect on p21 localization in normally growing cells (Fig 2 and S3A Fig). In contrast, SENP2 depletion in the absence of DNA insults produced a decrease in the number of cells with cytoplasmic HA-p21, suggesting that a lack of deSUMOylation impairs the cytoplasmic localization of p21 (Fig 2 and S3A Fig). Of note, depletion of either UBC9 or SENP2 did not disrupt the nucleoli in the absence of DNA damage and also did not affect Adr-induced nucleolar disruption as indicated by the presence of peripheral (segregated) fibrillarin and capped UBF in the nucleolus (S3B Fig).

Last, since p21 transits through the nucleolus [25], its localization was regulated by SUMO-1 conjugation and SUMO-1 was suggested to regulate the ribosome biogenesis, we tested if p21 has any impact directly in ribosome formation. When we measure the total protein synthesis or the rRNA processing in the absence of p21, we did not observe changes comparing to control cells, suggesting that p21 does not play a direct role ribosome biogenesis in normally growing cells (S4 Fig).

### Both p21 and SUMOylation favour INoB biogenesis upon DNA damage

**Fig 3:**
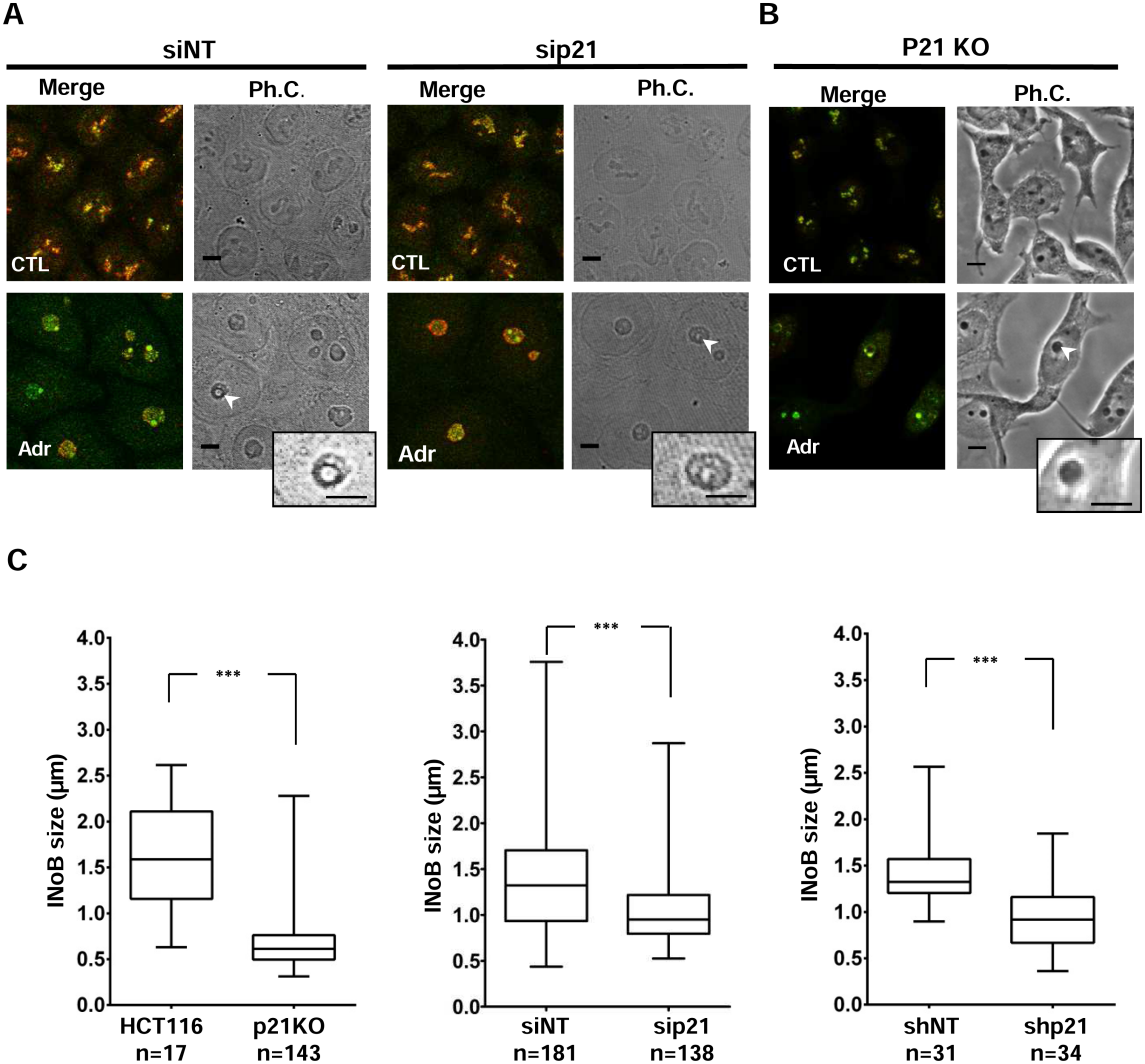
INoB growth is dependent on p21 whereas nucleolar disruption does not. **A)** Immunostaining of UBF (green) and Fibrillarin (red) to analyse nucleolar disruption HCT116 cells transfected with non-targeting (siNT), p21 (sip21) siRNAs not treated (CTL) or treated with Adr for 48 hours (Adr). Ph.C.: Phase contrast. Scale bar: 5μm. **B)** Immunostaining of UBF (green) and Fibrillarin (red) to analyse nucleolar disruption in HCT116 p21KO cells after Adr treatment for 48 hours versus control (CTL). Ph.C.: Phase contrast. Scale bar: 5μm. **C)** Box-plot graphs showing the INoB size (μm) in HCT116 cells versus HCT116 p21KO cells (left), in HCT116 cells transfected with non-targeting (siNT) versus p21 (sip21) siRNA (middle); and in HCT116 cells transfected with pSUPER-puro-EGFP (shNT) versus pSUPER-puro-EGFP-p21 (shp21) (right). All cells were treated with Adr for 48 hours. Box shows Median and first quartiles, and whiskers show Min and Max. Number of cells (n) for each condition is shown. Example of how INoB size is quantified is shown in S5A Fig.

**Fig 4:**
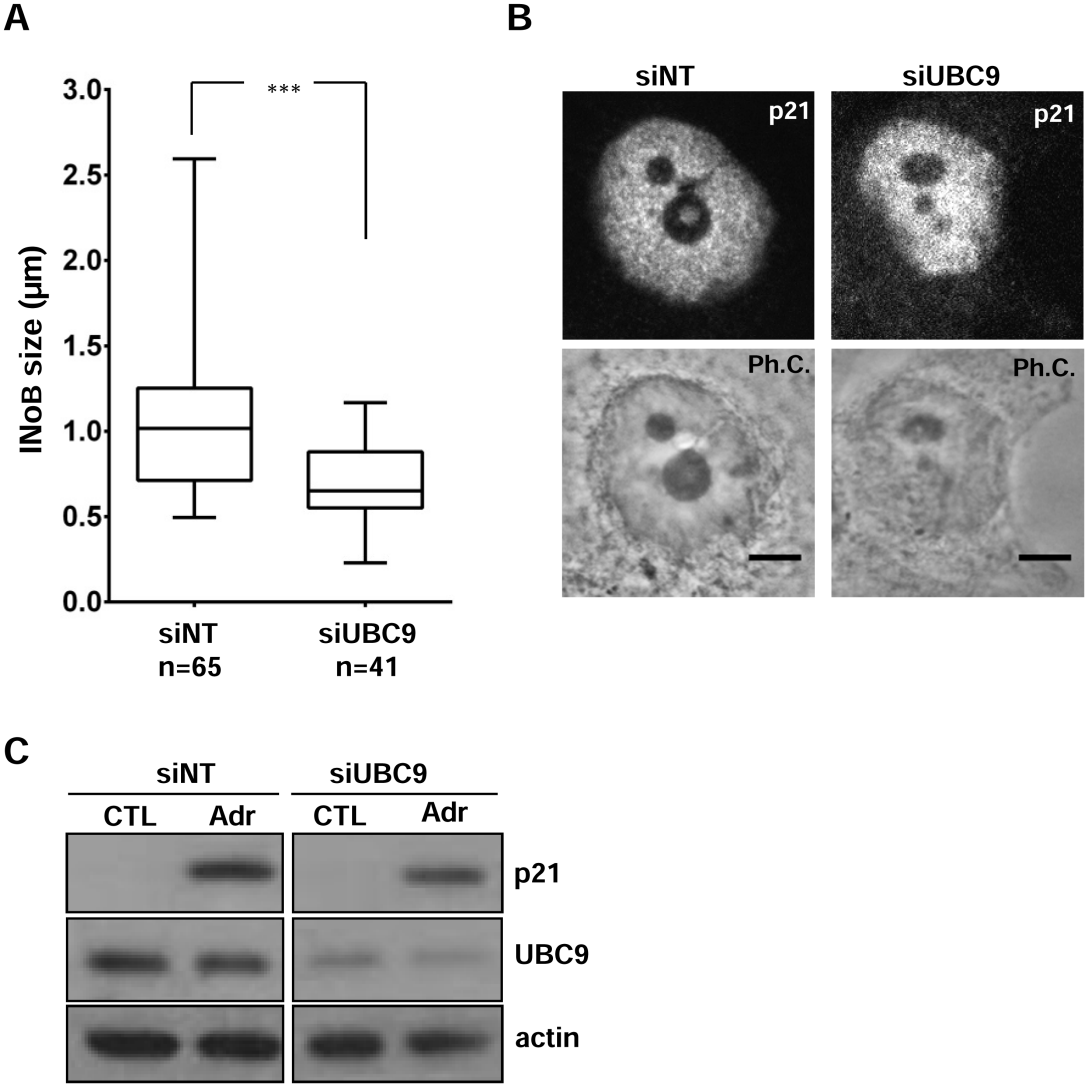
INoB size is reduced after UBC9 depletion. A) Box-plot graph showing the INoB size (μm) in HCT116 cells transfected with non-targeting (siNT) or UBC9 (siUBC9) siRNAs. All Cells were treated with Adr the last 24 hours. Box shows Median and first quartiles, and whiskers show Min and Max. Number of cells (n) for each condition is shown. B) Immunostaining of p21 in in HCT116 cells transfected with non-targeting (siNT) or UBC9 (siUBC9) siRNAs and treated with Adr for 24 hours. Ph.C.: Phase contrast. Scale bar: 5μm. C) Western blot analysis of p21 levels in HCT116 cells treated as in (A); actin was used as loading control.

Next, we explored the involvement of p21 and SUMO in the nucleolar disorganization and INoB formation induced by DNA damage. As shown for UBC9 and SENP2 (S3A Fig), p21 was not necessary for nucleolar disruption after Adr treatment, which was demonstrated in both HCT116 cells lacking p21 through siRNA depletion and p21-knockout HCT116 cells (HCT116 p21KO cells) (Fig 3A and 3B and S5 Fig). Furthermore, p21 was not involved in initiating INoB formation since the percentage of cells with INoBs was approximately 30% in all Adr-treated HCT116 cells after 24 hours, independent of p21 depletion. However, p21-depleted cells showed significantly smaller Adr-generated INoBs compared to those with normal p21 levels as measured by phase contrast analysis (Fig 3C and S5 Fig). This was also observed in HCT116 cells transfected with short hairpin RNA against p21 (Fig 3C and S5E Fig). Similar effects were seen with HCT116 cells treated with UBC9 siRNA. Interfering RNA against UBC9 did not change the percentage of INoB-positive cells after Adr (30%), but they had significantly smaller INoBs compared to siRNA control treated cells, demonstrating a role of SUMOylation in INoB size (Fig 4A). Accordingly, a concomitant decrease in nucleolar p21 accumulation was observed upon depletion of UBC9 (Fig 4B). Of note, total p21 levels upon Adr treatment were also high UBC9-depleted cells, indicating that UBC9 depletion effect in INoB size was not due to a lack of p21 accumulation (Fig 4C).

Recovery of the INoB size by p21 re-expression was analyzed in HCT116 p21KO cells (S6 Fig). Although p21 overexpression significantly increased INoB size, this was still smaller than that of HCT116 cells with normal p21 levels. Interestingly, SUMO overexpression in these Adr-treated cells increased INoB size. Adr-induced INoBs in HCT116 p21KO cells overexpressing both SUMO and p21 were of the same size as those produced in wild-type HCT116 cells (S6A Fig). Surprisingly, endogenous UBC9 expression was reduced in HCT116 p21KO cells compared to HCT116 cells, which would explain why these cells require SUMO overexpression to fully recover the INoB size (S6B Fig). To ascertain that the recovery of INoB size in HCT116 p21KO cells was not due to overexpressing any unspecific protein in the nucleolus, CFP-Cdk2, was overexpressed in these cells. As shown in S6A Fig, overexpression of CFP-Cdk2 did not increase INoB size when compared to non-transfected cells. The results indicated that protein SUMOylation and p21 are not associated with nucleolar disruption, but are involved in controlling INoB development upon Adr treatment.

### SUMO and p21 are essential for the accumulation of diverse DNA maintenance and chromatin organization proteins in INoBs following DNA damage

Since diverse DNA maintenance and chromatin organization proteins have shown to be localized in intranucleolar structures in a proportion of non-stressed cells [33], we asked whether their accumulation in INoBs following DNA damage was dependent on SUMO-1 and p21.

**Fig 5:**
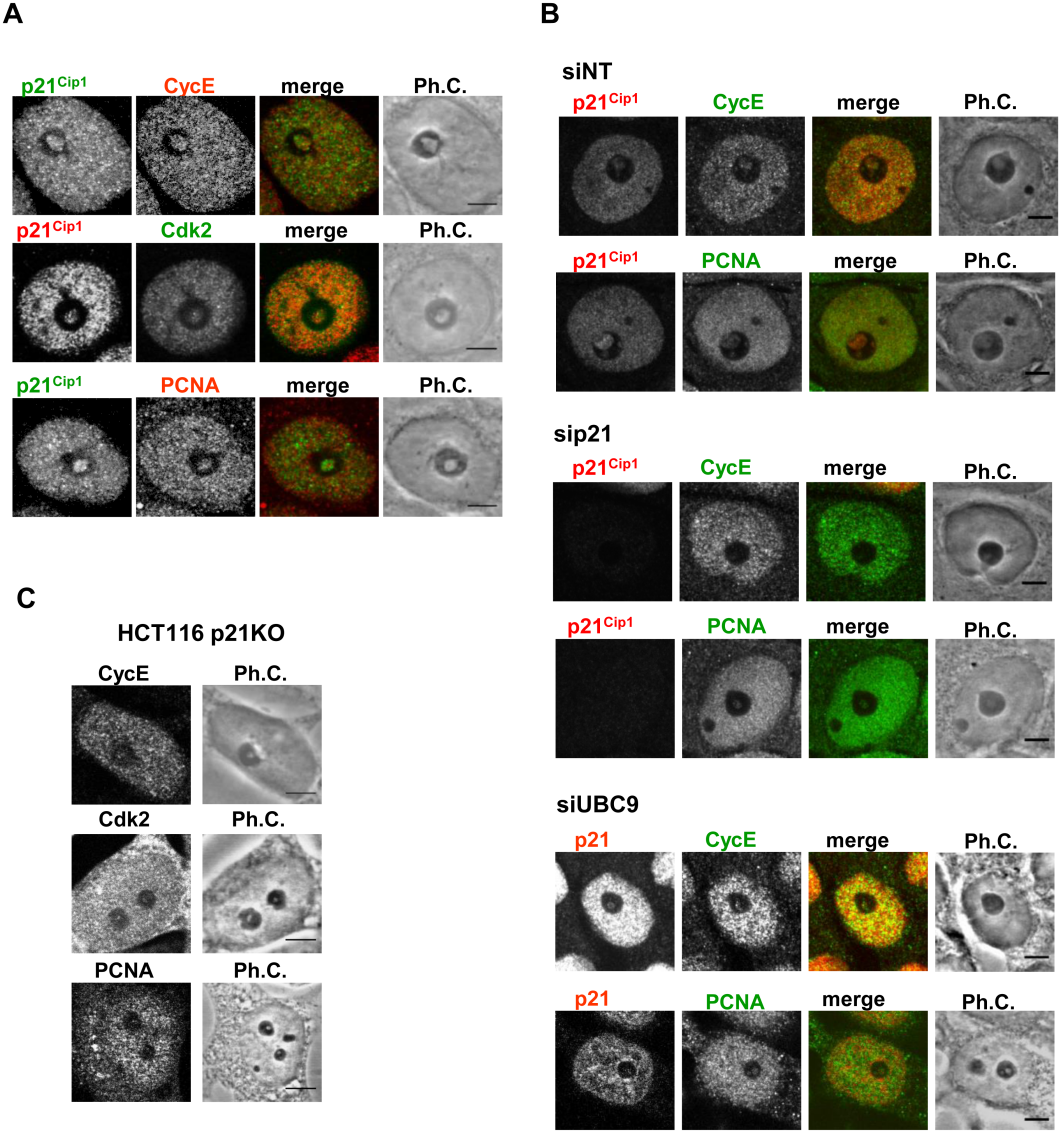
Cyclin E, PCNA and Cdk2 colocalize with p21 in the nucleolus in HCT116 cells after DNA damage. A) Immunostaining of endogenous p21 and CycE, PCNA or Cdk2 in HCT116 cells treated with Adr for 48 hours. B) Immunostaining of p21 and PCNA, CycE or Cdk2 HCT116 cells transfected with non-targeted (siNT), p21 (sip21) or UBC9 (siUBC9) siRNAs and treated with Adr for 48 hours. C) Immunostaining of PCNA, CycE or Cdk2 in HCT116 p21KO cells treated with Adr for 48 hours. Ph.C.: Phase contrast. Scale bar: 5μm.

We first analyzed the presence of Cyclin E, Cdk2 and PCNA, all p21-binding proteins. Cyclin E has already been reported to be present in the nucleolus, with Cdk2/Cyclin E kinase activity proposed to be involved in maintaining nucleolar functions [53,54]. Both endogenous Cyclin E and Cdk2 localized in the same nucleolar compartment as p21 in response to Adr treatment (Fig 5A). PCNA, a protein involved in DNA replication and repair, also colocalized with p21 in the nucleolus following DNA damage (Fig 5A). Interestingly, the smaller INoBs induced by DNA in cells with low levels of p21 or UBC9, also had decreased levels of Cyclin E and PCNA (Fig 5B and 5C and S7A Fig). Accordingly, when p21 and SUMO were exogenously expressed in HCT116 p21KO cells, PCNA, Cdk2, and Cyclin E levels in INoBs after DNA damage were restored (S7B Fig).

**Fig 6:**
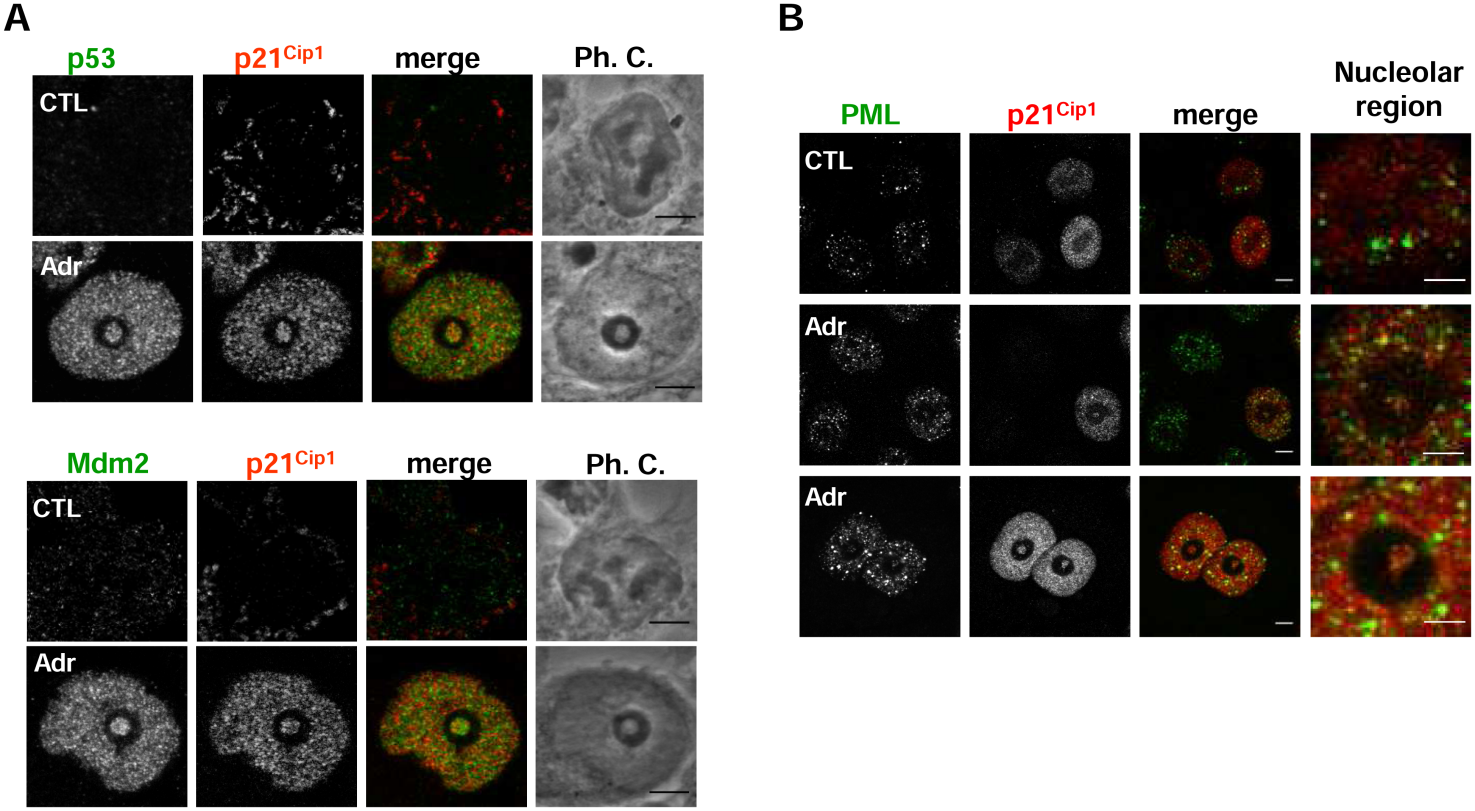
p53, Mdm2 and PML colocalize with p21 in the INoB. A) Immunodetection of endogenous p21 and p53 or Mdm2 in control (CTL) or HCT116 cells treated with Adr for 48 hours (Adr). Ph.C.: Phase contrast. Scale bar: 5μm. B) Immunodetection of endogenous p21 and PML in HCT116 cells untreated (CTL) or treated with Adr for 24 hours (Adr). Scale bar: 5μm

Nucleolar localization of p53 and Mdm2 was also analyzed since these proteins are linked to the DNA damage checkpoint response, they have already been described to localize at the nucleolus [9,55,56] and can also be SUMOylated [57,58]. Interestingly, both p53 and Mdm2 localized in INoBs following DNA damage (Fig 6A) and their nucleolar accumulation was reduced under conditions that produced smaller INoBs, e.g., in HCT116 p21KO cells or UBC9-depleted cells (S8A and S8B Fig). The presence of the promyelocytic leukemia (PML) protein in INoBs was also analyzed since it is a major target for covalent modification by SUMO, and it contains SUMO-interacting-motifs (SIM) in its sequence, which allows PML to act as a scaffold protein for the formation of PML-nuclear bodies (PML-NB) and for chromatin organization [59]. Furthermore, it has also been shown to translocate to the nucleolus following DNA damage [60]. We observed PML1 in INoBs, together with p21, in Adr-treated HCT116 cells (Fig 6B).

**Fig 7:**
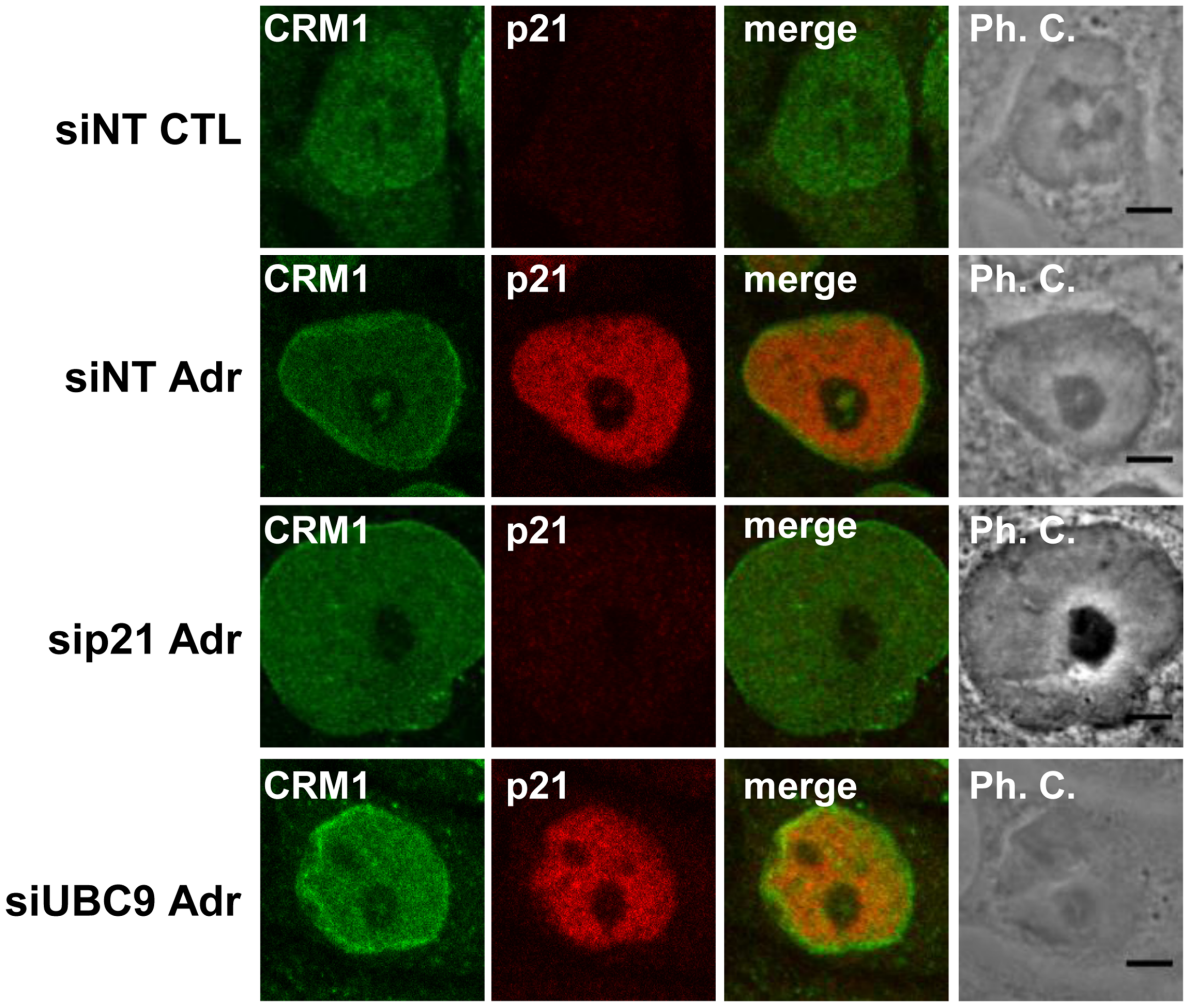
CRM1 colocalize with p21 in the INoB. Immunodetection of endogenous CRM1 and p21 in HCT116 cells transfected with non-targeting (siNT), p21 (siP21) or UBC9 (siUBC9) siRNAs. Cells were non-tretaed (CTL) or treated with Adr for 24 hours (Adr). Ph.C.: Phase contrast. Scale bar: 5μm.

Finally, we determined whether CRM1 accumulated in INoBs upon DNA damage in a p21- and SUMOylation- dependent manner. CRM1 is an exportin that is associated with the translocation of several proteins from the nucleus to the cytoplasm, including, p21, p53, and Mdm2 [61,62], as well as specific ribosomal proteins [17]. CRM1 also regulates the nucleolar localization of proteins involved in ribosome biogenesis [63]. Furthermore, Ernoult-Lange *et al* (2009) [64] reported that impaired ribosome biogenesis elicited CRM1 accumulation in the nucleoli in specific foci that they termed CRM1 nucleolar bodies. Our data show that CRM1 had a diffuse nuclear localization in control HCT116 cells, but accumulated in the nuclear envelope and INoBs following DNA damage, where it colocalized with p21 (Fig 7). Moreover, CRM1 accumulation in these nucleolar structures significantly dropped in p21- or UBC9–depleted cells (Fig 7 and S8C Fig).

Taken together theses data indicate that p21, SUMO, and protein SUMOylation are important for INoB assembly after DNA damage, as well as for the accumulation of p21-binding proteins (Cyclin E, PCNA, and Cdk2), DNA damage checkpoint proteins (p53 and Mdm2), SUMO-interacting proteins (PML1), and the exportin CRM1 in INoBs.

## Discussion

We have previously shown that upon DNA damage and other types of nucleolar stress, p21-containing INoBs form in a great variety of cell lines [25]. Here, we report that endogenous SUMO-1 also accumulates in INoBs. Moreover, while exogenous wild type SUMO-1 accumulates in INoBs, mutant SUMO-1 that is unable to conjugate with proteins does not. Thus, our data show that SUMOylated proteins accumulate in INoBs together with p21 following DNA damage. The presence of UBC9, the only known E2 SUMO-conjugating enzyme, in these structures suggests that SUMOylation may be actively occurring there. To the best of our knowledge, this is the first time that UBC9 has been reported to be present in the nucleolus. Since SENP3 and 5, two SUMO isopeptidases, have also been observed in the nucleolus [65,66], it is likely that protein SUMOylation and deSUMOylation occur in the nucleolus. Our results also indicate that both p21 and SUMOylation are essential for INoB biogenesis and the concomitant nucleolar accumulation of diverse p21- and SUMO-interacting proteins that are essential for cell cycle regulation, DNA damage checkpoints and chromatin organisation.

We previously showed that p21 transits through the nucleolus during its passage from the nucleus to the cytoplasm [25]. Here, we demonstrate that SUMOylation regulates the distribution of p21 between the nucleus and the cytoplasm. Cytoplasmic p21 has an oncogenic role and has been linked to some breast tumours [30] and lymphomas [67], while nuclear p21 inhibits cell proliferation and acts as a tumour suppressor [31]. Interestingly, inhibition of nuclear export is becoming an important and promising target in cancer therapy [68]. Meanwhile, SUMOylation has been shown to regulate the transit of proteins through the nuclear pores [69], with SENP2, a deSUMOylation enzyme, being observed at nuclear pores [36]. We propose that SENP2 inhibition could be used to decrease cytoplasmic p21 localization and, consequently, promote its tumour suppressive role.

We had postulated that the transit of p21 through the nucleolus in non-stress conditions was essential for regulating ribosomal biogenesis. However, our data on protein synthesis and rRNA synthesis and processing do not support this hypothesis.

In addition to covalently conjugating with proteins, SUMO-1 can also interact with a variety of proteins containing SIMs, thereby promoting the creation of multiprotein complexes [40]. The interaction of SUMO-1 with the SIM of PML is essential for the formation of PML bodies [59]. Moreover, p21 can interact with a variety of proteins either through its N-terminal globular domain or its C-terminal domain, which is flexible and can interact with a diverse range of proteins [70,71]. Since we observed p21- and SUMOylation-dependent accumulation of several p21-binding proteins (PCNA, Cdk2, and Cyclin E) and SUMO-conjugated proteins (p53, Mdm2, and PML1) in INoBs following DNA damage, we propose that p21 and SUMO act as “hub” proteins, creating a nucleation platform to increase the concentration of specific proteins in the nucleolus. This would facilitate interactions that are important for the stress response. In this sense, SUMOylated PCNA might have a function in rDNA repair by homologous recombination [72] and p53 binds to TATA-binding proteins and TBP–associated factor 1 of the SL-1 complex, preventing its interaction with UBF and thus formation of transcription-competent Pol I complex [73]. In agreement with the formation of these platforms, we also noticed PML colocalization with p21 in INoBs. This is in agreement with an earlier study showing that nucleolar PML following DNA damage was critical for Mdm2 nucleolar localization and p53 stabilization [60].

Alternatively, although not mutually exclusive with the abovementioned model, the accumulation of some of the proteins in the INoBs may be due to DNA damage disrupting their normal translocation through the nucleolus to the cytoplasm or their shuttling between the nucleolus and the nucleoplasm, thus trapping the proteins in the INoBs. Indeed, we observed the accumulation in the INoBs of CRM1, an exportin required also to transport proteins into the nucleolus [63]. Accordingly, nucleolar CRM1 accumulation has already been described in response to RNA polymerase I inhibition [64]. The authors of that study proposed that a platform loading CRM1 onto pre-60S ribonucleoprotein particles occur in nucleoli (CNoB) in normal conditions, which, upon nucleolar disruption, causes further nucleolar CRM1 accumulation. Here, we show a similar effect in response to Adr treatment. However, we propose that the export of other proteins using the same ribonucleoprotein particle export route is also blocked. Our results indicate that p21[74] and SUMOylation are important for the transit of these proteins through the nucleolus, since the depletion of p21 or UBC9 prevented their nucleolar accumulation following DNA damage. One possibility is that p21 and SUMOylation facilitate the loading of CRM1 on to NES-containing proteins entering the nucleolus, therefore playing an essential role in nucleoplasm-nucleolus shuttling and nuclear export through the nucleolus. The transit of some of these proteins through the nucleolus under non-stress conditions could be linked to the fundamental role of the nucleolus in monitoring and responding to different cellular stress signals that affect ribosome biogenesis [21]. Two export routes from the nucleus to the cytoplasm have been proposed for p53, one through the nucleolus, where the protein is polyubiquitinated and labeled for degradation in the cytoplasm, and another through the nucleoplasm, where the protein is monoubiquitinated and sent to the cytoplasm to perform specific functions such as inhibiting apoptosis [10]. Blocking the export from the nucleolus by nucleolar disruption facilitates p53 accumulation in the nucleus, where it may play an important role in the stress response. Furthermore, proteasome-independent, Def- and calpain 3b-dependent degradation of p53 in the nucleolus has been described in non-stress conditions [75], while Cyclin E has also been shown to be polyubiquitinated and tagged for degradation in the nucleolus [76].

We propose that p21 and SUMO are involved in the active accumulation of p21- and SUMO-interacting proteins in the nucleolus following DNA damage to facilitate their specific functions in the stress response. Alternatively, they could be associated with regulating the transit of these proteins through the nucleolus under control conditions, which is impeded following DNA damage.

Dysregulated ribosome biogenesis is required for the survival of malignant cells, thus making nucleolar activity an important therapeutic target in the treatment against cancer [77,78]. The data presented here will help to reveal the mechanisms involved in regulating the transit and accumulation of proteins in the nucleolus and might contribute to the design of new cancer therapies.

## Supporting information

S1 Fig Transcription is inhibited in nucleoli with INoB. Immunodetection of endogenous p21 and incorporated 5’FU in HCT116 cells treated with Adr for 24 hours and recovered in the absence of Adr for 24 hours. 5’FU was added during the last 15 min. Arrow indicates a cell without INoB and positive for 5’FU incorporation in the nucleolus, while arrowhead indicates a cell with INoB and negative for 5’FU incorporation in the nucleolus. Scale bar: 10μm. Graph shows mean intensity (arbitrary units) quantification of 5’FU incorporation in the nucleolus in p21 positive cells comparing INoB positive versus INoB negative cells. Number of cells (n) analysed for each condition is shown. Box shows Median and first quartiles, and whiskers show Min and Max.
(TIF)

S2 Fig SUMO-1 colocalization with p21 in the INoB using anti-SUMO-1 rabbit antibodies. Immunodetection of endogenous SUMO-1 (green) using anti-SUMO rabbit antibody and p21 (red) using anti-p21 mouse antibody in HCT116 control cells (CTL) or treated with Adr for 48 hours (Adr). Scale bar: 5μm. B) Quantification of SUMO-1 immunostaining (integrated density) in INoBs of 24-h Adr-treated HCT116 cells transfected with non-targeting (siNT) or UBC9 (siUBC9). Number of cells (n) analysed for each condition is shown. Box shows Median and first quartiles, and whiskers show Min and Max.
(TIF)

S3 Fig Effect of UBC9 and SENP2 depletion on HA-p21 intracellulaar localization and nucleolar organization. A) Representative images of HA-p21 intracellular localization in cells used for the quantification shown in Fig 2. In the upper panels the most frequent phenotypes are shown. The specific frequencies (%) of each phenotype are indicated in each image. Scale bar: 5μm. The arrows indicate InoBs magnified in the inserts. B) Immunostaining of UBF (fibrillar center marker) and Fibrillarin (dens fibrillar component marker) upon transfection of HCT116 cells with non-targeting (siNT), SENP-2 (siSENP2) or UBC9 (siUBC9) siRNAs. Cells were non treated (CTL) or treated with Adr for 24 hours (Adr). Ph.C.: Phase contrast. Scale bar: 5μm.
(TIF)

S4 Fig p21 depletion does not affect rRNA processing nor the novo protein synthesis. A) Ethidium Bromide-stained agarose gel (left) and autoradiogram of a northern blot (middle) of total cellular RNA from non-targeting (siNT), p21 (sip21), UBC9 (siUBC9), SENP2 (siSENP2) or S6 (siS6) siRNAs transfected HCT116 cells during 48h. Newly synthesised RNA was pulse labelled with ^3^H-Uridine for 1h and then was chased for 4h in non-labelled uridine-containing medium; 1μg of total cellular RNA was loaded per lane. Western blot (right) showing the levels of p21, UBC9, SENP2 and S6 upon the different siRNA transfections. S6 depletion was used as positive control of rRNA synthesis inhibition. B) Left: Graph showing quantification of ^3^H-Leucine incorporation into proteins, in HCT116 cells transfected with non-targeting (siNT), p21 (sip21) or S6 (siS6) siRNAs, and of HCT116 cells treated with 100 μg/ml chycloheximide (CHX) for 10 minutes prior to ^3^H-leucine incorporation; right: Western blot showing the levels of p21 and S6 upon the different siRNA transfections. Actin was used as loading control. Chycloheximide treatment and S6 depletion were used as positive controls of protein synthesis inhibition.
(TIF)

S5 Fig Images and controls related to Fig 3. A) Example of how INoB size was quantified using the Image J programme. First, the phase contrast image was magnified and scaled. Then, a line was draw through the maximum INoB dimension and its length was measured by the Image J program. When a nucleolus had more than one INoB the bigger one was measured. The percentage of nucleolus with multi INoBs was similar in all treatments. B) Quantification of INoB size in phase contrast images of HCT116 cells transfected with p21 (sip21) siRNA and treaded with Adr. Immunostaining of p21 was performed and INoB size of cells with real depletion of p21 (p21 negative cells) versus cells with low depletion of p21 (p21 positive cells) is shown in the graph. To see examples of the quantified cells see panel Adr-treated cells in (C). Number of cells (n) analysed for each condition is shown. Box shows Median and first quartiles, and whiskers show Min and Max. C) Example of p21 immunostaining and phase contrast images of HCT116 cells transfected with p21 (sip21) or non-targeted (siNT) siRNA and treated with Adr. D) Western blots showing p21 levels of cells HCT116 cells transfected with non-targeting (siNT) or p21 (sip21) siRNAs and in HCT116 and HCT116 p21KO (p21KO) cells. Cells were non treated (CTL) or treated with Adr for 24 hours (Adr). Actin was used as loading control. E) Immunostaining of p21 (red) and GFP visualization (green) of HCT116 cells transfected with pSUPER-puro-EGFP-p21 (shp21). Ph.C.: Phase contrast. Scale bar: 5μm.
(TIF)

S6 Fig Recovery of INoB growth in HCT116 p21KO cells by expression of p21 and SUMO-1. A) Box-blot graph of INoB size measurement in HCT116 p21KO cells after transfection with GFP-p21 and/or Orange-SUMO-1, or CFP-Cdk2. A representative image is shown for each condition. Arrows in the phase contrast images indicated transfected cells. Ph.C.: Phase contrast. Scale bar: 5μm. Box shows Median and first quartiles, and whiskers show Min and Max. ns: non-significant differences. Number of cells (n) analysed for each condition is shown. B) Western Blots showing using SUMO-1 and UBC9 antibodies of lysates from HCT116 and HCT116 p21KO cells (p21KO), non-treated (CTL) or treated with Adr (Adr) for the indicated time (h: hours). Actin was used as loading control.
(TIF)

S7 Fig Nucleolar localization of CycE, Cdk2 and PCNA is p21 and SUMOylation dependent. A) Graph showing the quantification of CycE, Cdk2 and PCNA immunostaining in INOBs (diameter of the fluorescence signal) in HCT116 cells transfected with non-targeting (siNT), UBC9 (siUBC9) or p21 (sip21) siRNAs, and treated with Adr for 48 hours. Box shows Median and first quartiles, and whiskers show Min and Max. Number of cells (n) analysed for each condition is shown. B) Immunostaining of endogenous CycE, Cdk2 and PCNA in HCT116 p21KO cells non-transfected (using a secondary antibody conjugated to Alexa488) or co-transfected with both GFP-p21 and Orange-SUMO (using a secondary antibody conjugated to Alexa647). Ph.C.: Phase contrast. Scale bar: 5μm
(TIF)

S8 Fig Localization of p53, Mdm2 and CRM1 in INoBs is dependent on p21 and UBC9. A) Immunostaining of p53 and MDM2 in HCT116 p21KO cells treated with Adr for 48 hours. Ph.C.: Phase contrast. Scale bar: 5μm. B) Immunostaining of p53 in HCT116 transfected with non-targeting (siNT) or UBC9 (siUBC9) siRNAs and treated with Adr for 24 hours. Ph.C.: Phase contrast. Scale bar: 5μm. C) Box plot graph showing CRM1 immunostaing in INoBs (diameter of the fluorescence signal) in HCT116 cells transfected with with non-targeting (sNT), p21 siRNA (sip21) or UBC9 siRNA (siUBC9) and treated with Adr for 24 hours. Box shows Median and first quartiles, and whiskers show Min and Max. Number of cells (n) analysed for each condition is shown.
(TIF)
