# Supplementary figures and images for: SUMO regulates p21Cip1 intracellular distribution and with p21Cip1 facilitates multiprotein complex formation in the nucleolus upon DNA damage
Source: PLoS One. 2017 Jun 5;12(6):e0178925. doi: 10.1371/journal.pone.0178925 (PMC5459497; doi:10.1371/journal.pone.0178925)

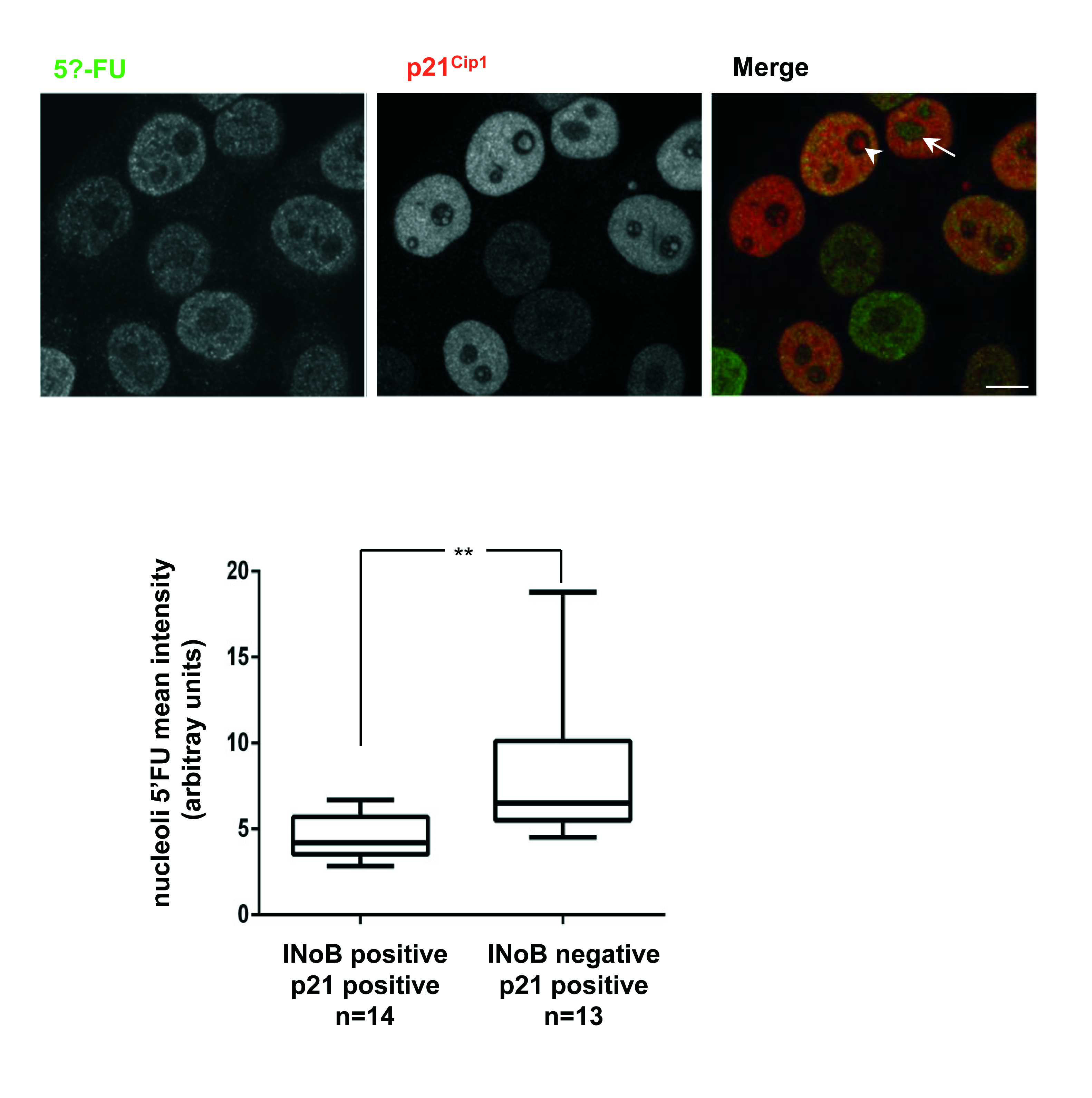

Supplement: S1 Fig — Immunodetection of endogenous p21 and incorporated 5’FU in HCT116 cells treated with Adr for 24 hours and recovered in the absence of Adr for 24 hours. 5’FU was added during the last 15 min. Arrow indicates a cell without INoB and positive for 5’FU incorporation in the nucleolus, while arrowhead indicates a cell with INoB and negative for 5’FU incorporation in the nucleolus. Scale bar: 10μm. Graph shows mean intensity (arbitrary units) quantification of 5’FU incorporation in the nucleolus in p21 positive cells comparing INoB positive versus INoB negative cells. Number of cells (n) analysed for each condition is shown. Box shows Median and first quartiles, and whiskers show Min and Max. (TIF) [file pone.0178925.s001.tif]

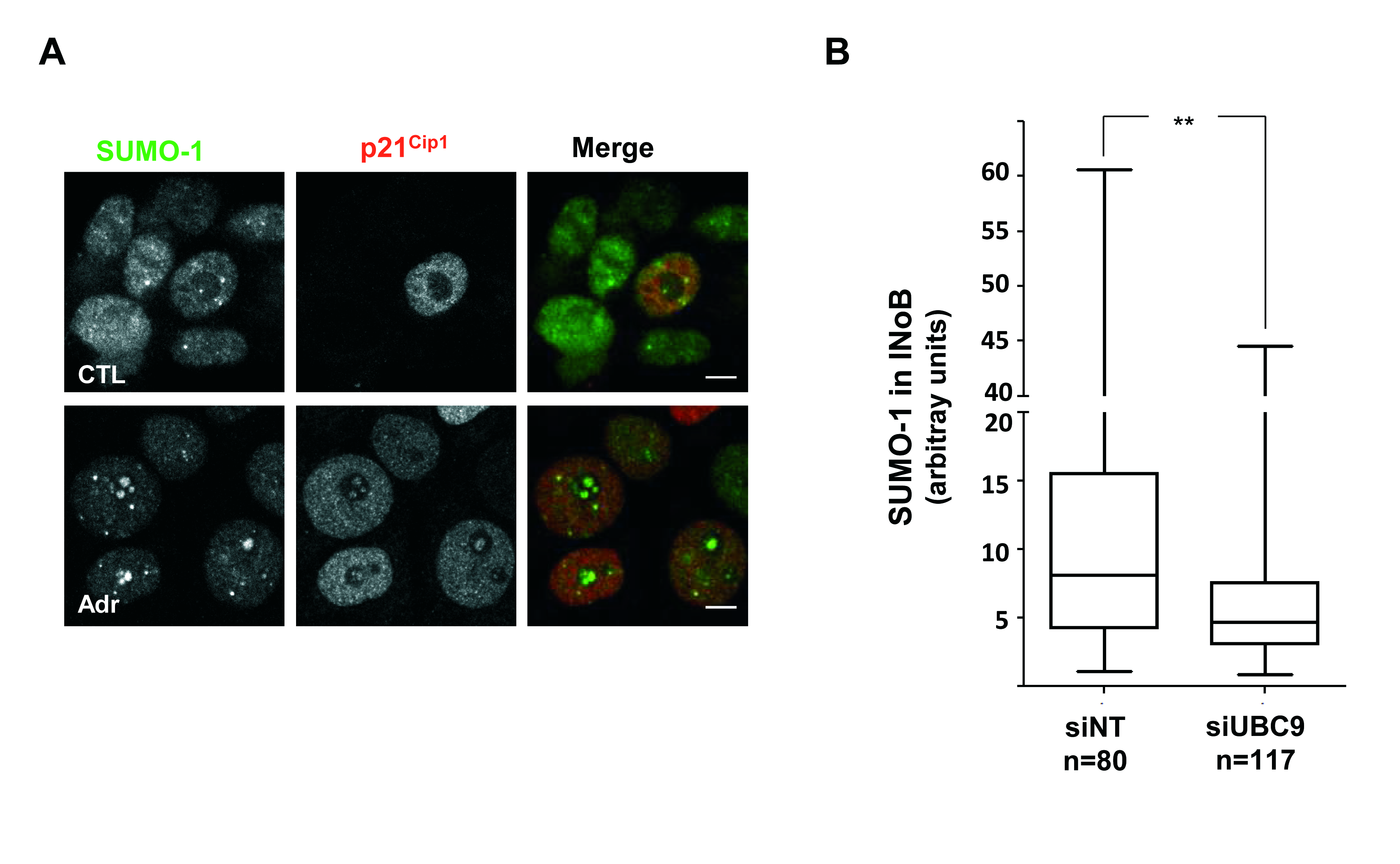

Supplement: S2 Fig — Immunodetection of endogenous SUMO-1 (green) using anti-SUMO rabbit antibody and p21 (red) using anti-p21 mouse antibody in HCT116 control cells (CTL) or treated with Adr for 48 hours (Adr). Scale bar: 5μm. B) Quantification of SUMO-1 immunostaining (integrated density) in INoBs of 24-h Adr-treated HCT116 cells transfected with non-targeting (siNT) or UBC9 (siUBC9). Number of cells (n) analysed for each condition is shown. Box shows Median and first quartiles, and whiskers show Min and Max. (TIF) [file pone.0178925.s002.tif]

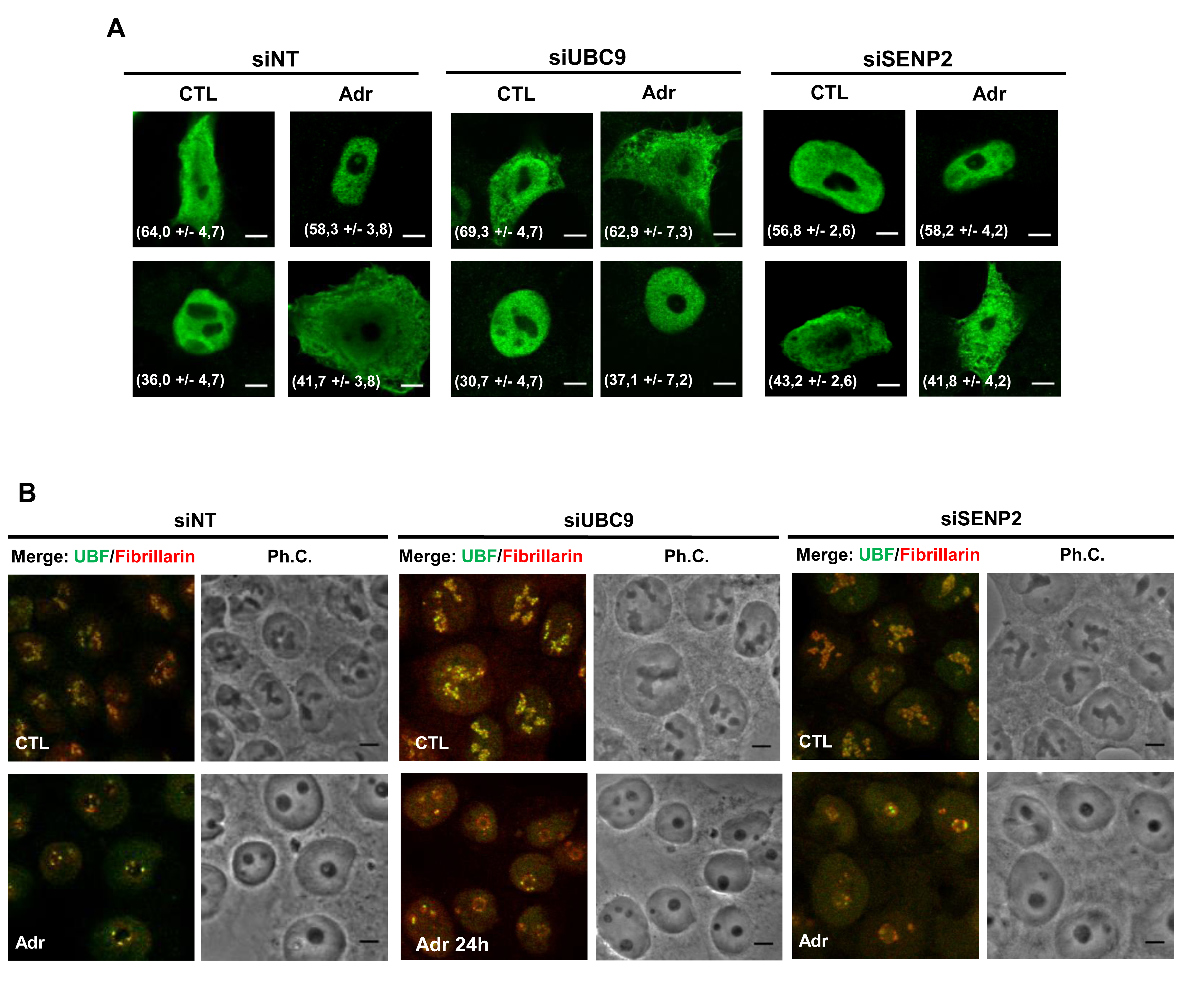

Supplement: S3 Fig — A) Representative images of HA-p21 intracellular localization in cells used for the quantification shown in Fig 2. In the upper panels the most frequent phenotypes are shown. The specific frequencies (%) of each phenotype are indicated in each image. Scale bar: 5μm. The arrows indicate InoBs magnified in the inserts. B) Immunostaining of UBF (fibrillar center marker) and Fibrillarin (dens fibrillar component marker) upon transfection of HCT116 cells with non-targeting (siNT), SENP-2 (siSENP2) or UBC9 (siUBC9) siRNAs. Cells were non treated (CTL) or treated with Adr for 24 hours (Adr). Ph.C.: Phase contrast. Scale bar: 5μm. (TIF) [file pone.0178925.s003.tif]

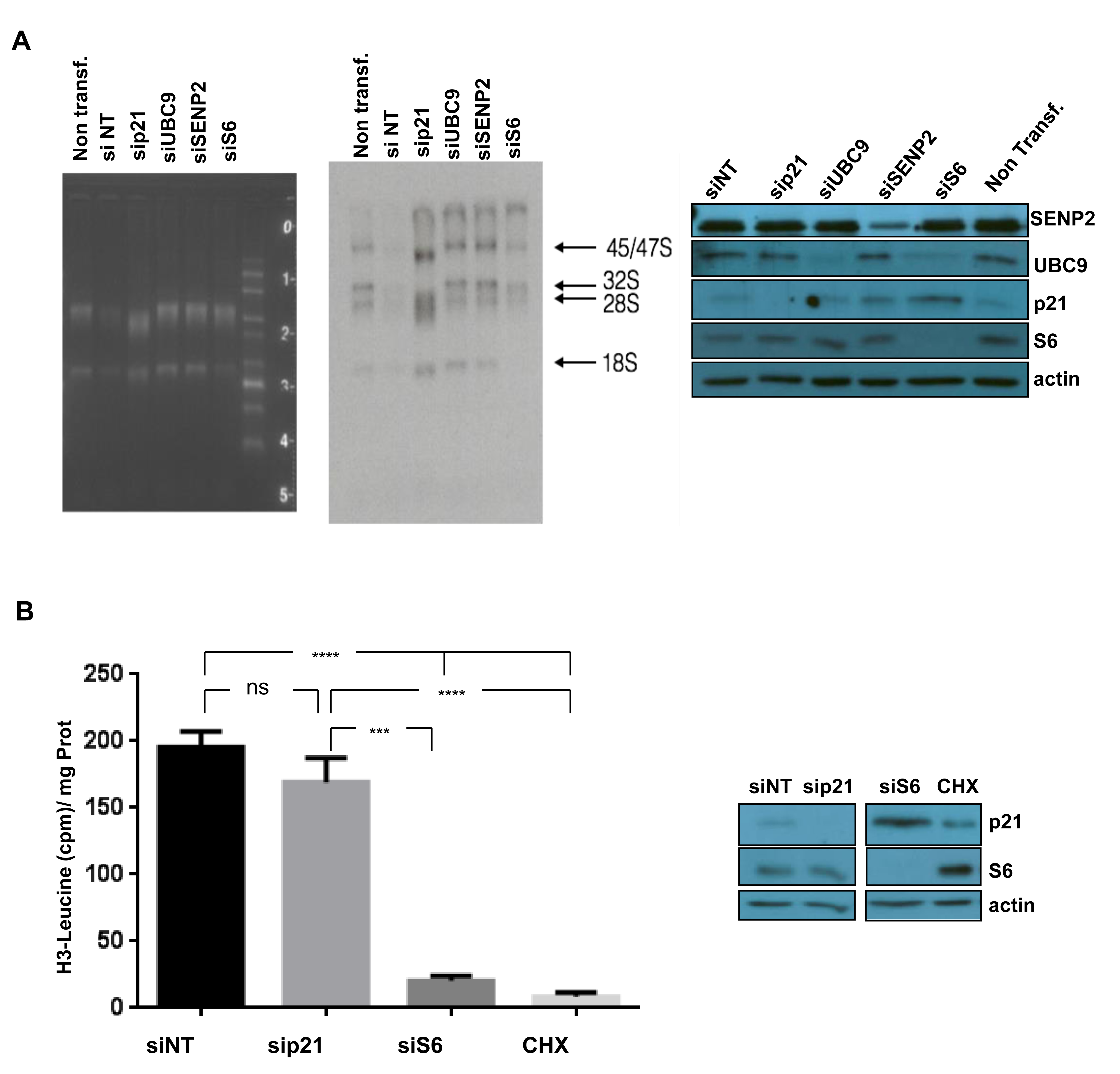

Supplement: S4 Fig — A) Ethidium Bromide-stained agarose gel (left) and autoradiogram of a northern blot (middle) of total cellular RNA from non-targeting (siNT), p21 (sip21), UBC9 (siUBC9), SENP2 (siSENP2) or S6 (siS6) siRNAs transfected HCT116 cells during 48h. Newly synthesised RNA was pulse labelled with 3H-Uridine for 1h and then was chased for 4h in non-labelled uridine-containing medium; 1μg of total cellular RNA was loaded per lane. Western blot (right) showing the levels of p21, UBC9, SENP2 and S6 upon the different siRNA transfections. S6 depletion was used as positive control of rRNA synthesis inhibition. B) Left: Graph showing quantification of 3H-Leucine incorporation into proteins, in HCT116 cells transfected with non-targeting (siNT), p21 (sip21) or S6 (siS6) siRNAs, and of HCT116 cells treated with 100 μg/ml chycloheximide (CHX) for 10 minutes prior to 3H-leucine incorporation; right: Western blot showing the levels of p21 and S6 upon the different siRNA transfections. Actin was used as loading control. Chycloheximide treatment and S6 depletion were used as positive controls of protein synthesis inhibition. (TIF) [file pone.0178925.s004.tif]

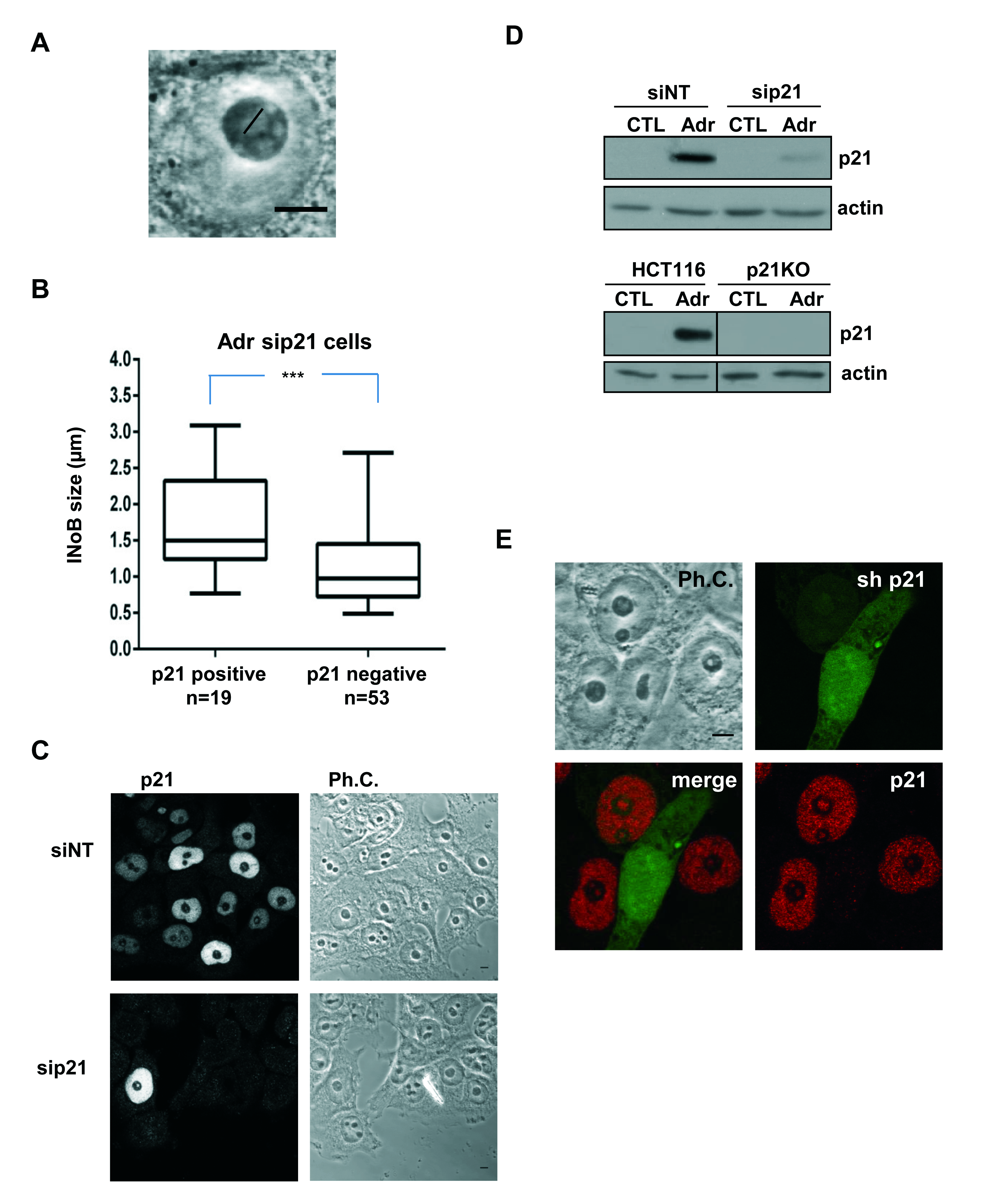

Supplement: S5 Fig — A) Example of how INoB size was quantified using the Image J programme. First, the phase contrast image was magnified and scaled. Then, a line was draw through the maximum INoB dimension and its length was measured by the Image J program. When a nucleolus had more than one INoB the bigger one was measured. The percentage of nucleolus with multi INoBs was similar in all treatments. B) Quantification of INoB size in phase contrast images of HCT116 cells transfected with p21 (sip21) siRNA and treaded with Adr. Immunostaining of p21 was performed and INoB size of cells with real depletion of p21 (p21 negative cells) versus cells with low depletion of p21 (p21 positive cells) is shown in the graph. To see examples of the quantified cells see panel Adr-treated cells in (C). Number of cells (n) analysed for each condition is shown. Box shows Median and first quartiles, and whiskers show Min and Max. C) Example of p21 immunostaining and phase contrast images of HCT116 cells transfected with p21 (sip21) or non-targeted (siNT) siRNA and treated with Adr. D) Western blots showing p21 levels of cells HCT116 cells transfected with non-targeting (siNT) or p21 (sip21) siRNAs and in HCT116 and HCT116 p21KO (p21KO) cells. Cells were non treated (CTL) or treated with Adr for 24 hours (Adr). Actin was used as loading control. E) Immunostaining of p21 (red) and GFP visualization (green) of HCT116 cells transfected with pSUPER-puro-EGFP-p21 (shp21). Ph.C.: Phase contrast. Scale bar: 5μm. (TIF) [file pone.0178925.s005.tif]

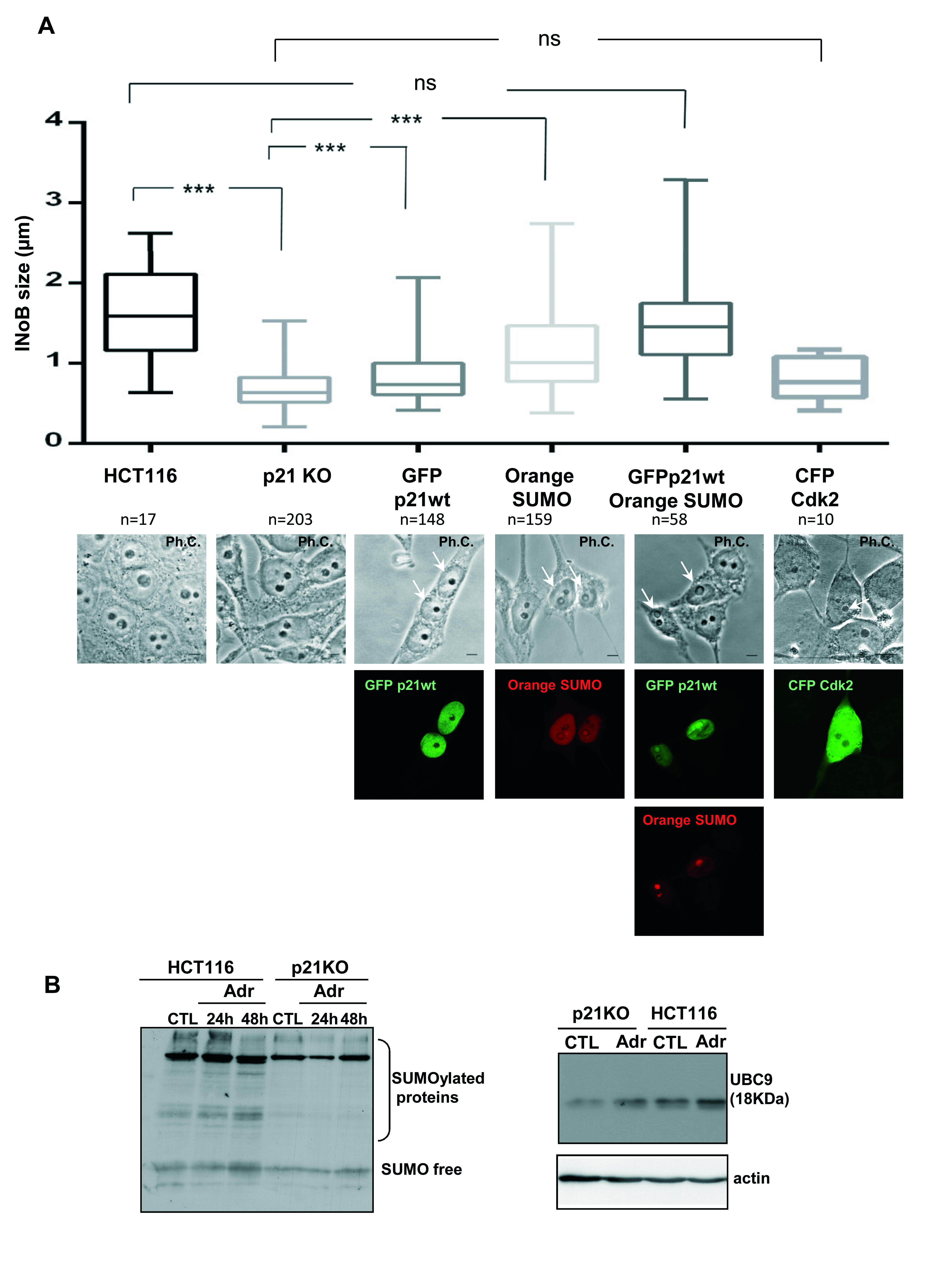

Supplement: S6 Fig — A) Box-blot graph of INoB size measurement in HCT116 p21KO cells after transfection with GFP-p21 and/or Orange-SUMO-1, or CFP-Cdk2. A representative image is shown for each condition. Arrows in the phase contrast images indicated transfected cells. Ph.C.: Phase contrast. Scale bar: 5μm. Box shows Median and first quartiles, and whiskers show Min and Max. ns: non-significant differences. Number of cells (n) analysed for each condition is shown. B) Western Blots showing using SUMO-1 and UBC9 antibodies of lysates from HCT116 and HCT116 p21KO cells (p21KO), non-treated (CTL) or treated with Adr (Adr) for the indicated time (h: hours). Actin was used as loading control. (TIF) [file pone.0178925.s006.tif]

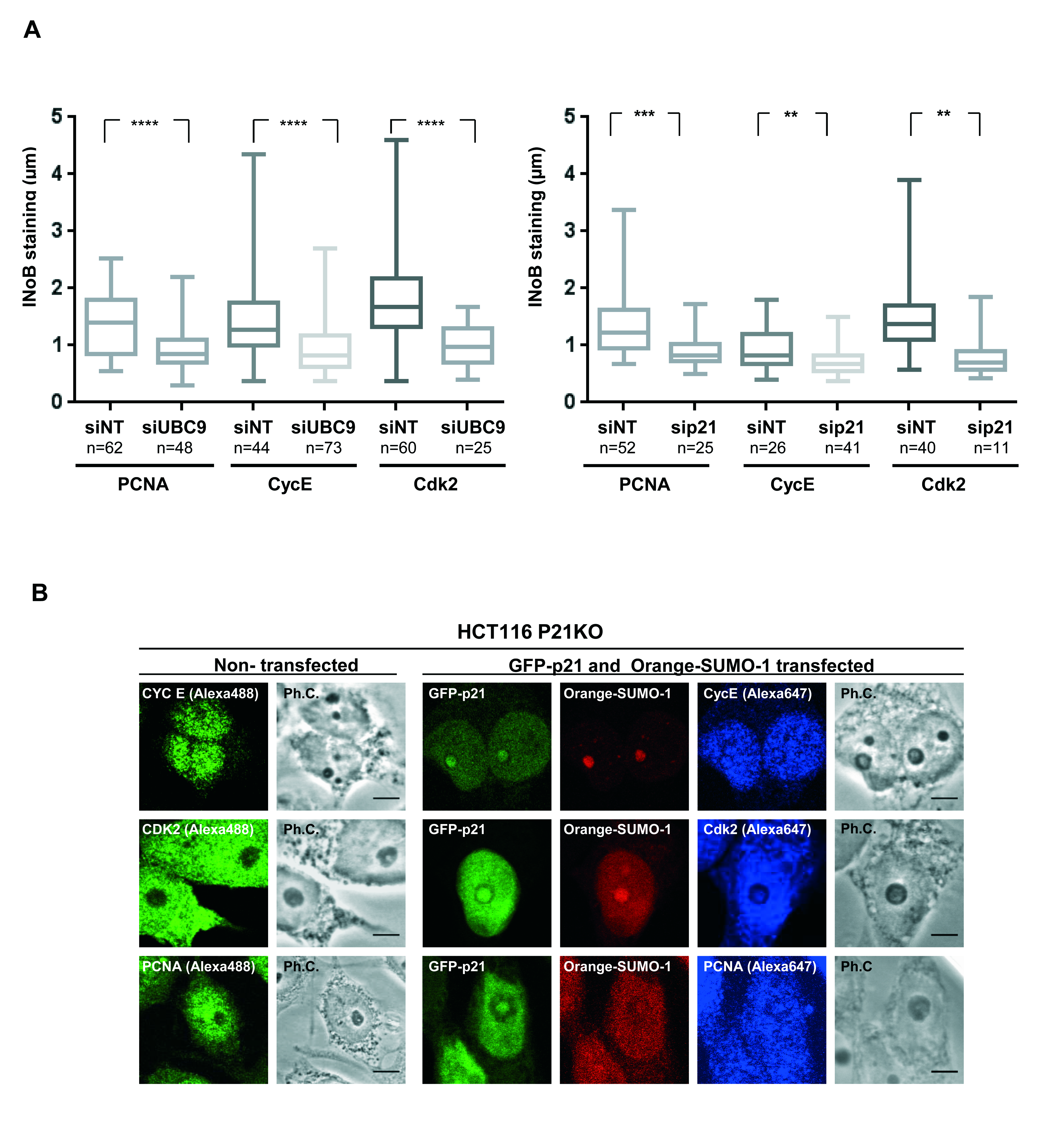

Supplement: S7 Fig — A) Graph showing the quantification of CycE, Cdk2 and PCNA immunostaining in INOBs (diameter of the fluorescence signal) in HCT116 cells transfected with non-targeting (siNT), UBC9 (siUBC9) or p21 (sip21) siRNAs, and treated with Adr for 48 hours. Box shows Median and first quartiles, and whiskers show Min and Max. Number of cells (n) analysed for each condition is shown. B) Immunostaining of endogenous CycE, Cdk2 and PCNA in HCT116 p21KO cells non-transfected (using a secondary antibody conjugated to Alexa488) or co-transfected with both GFP-p21 and Orange-SUMO (using a secondary antibody conjugated to Alexa647). Ph.C.: Phase contrast. Scale bar: 5μm (TIF) [file pone.0178925.s007.tif]

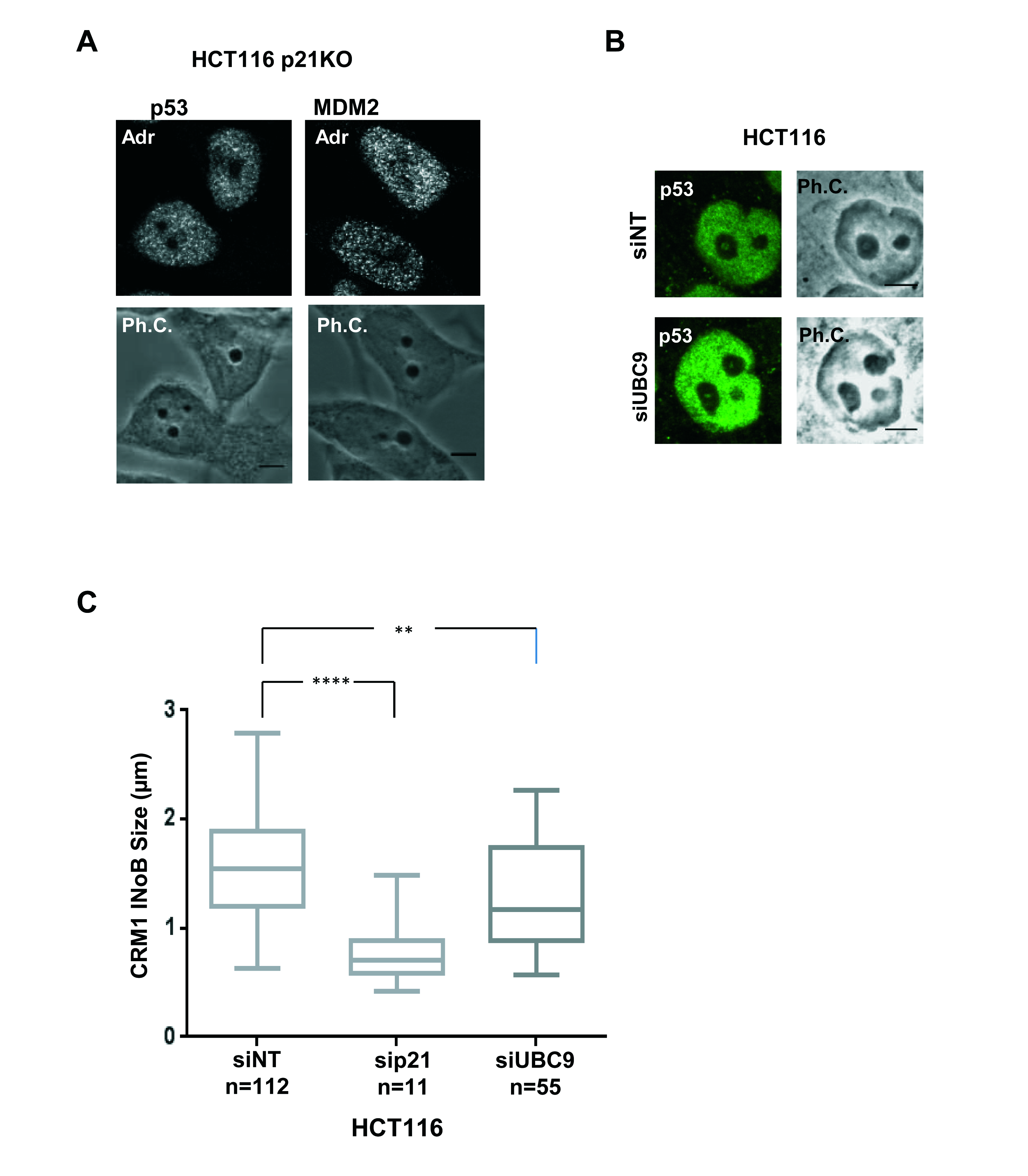

Supplement: S8 Fig — A) Immunostaining of p53 and MDM2 in HCT116 p21KO cells treated with Adr for 48 hours. Ph.C.: Phase contrast. Scale bar: 5μm. B) Immunostaining of p53 in HCT116 transfected with non-targeting (siNT) or UBC9 (siUBC9) siRNAs and treated with Adr for 24 hours. Ph.C.: Phase contrast. Scale bar: 5μm. C) Box plot graph showing CRM1 immunostaing in INoBs (diameter of the fluorescence signal) in HCT116 cells transfected with with non-targeting (sNT), p21 siRNA (sip21) or UBC9 siRNA (siUBC9) and treated with Adr for 24 hours. Box shows Median and first quartiles, and whiskers show Min and Max. Number of cells (n) analysed for each condition is shown. (TIF) [file pone.0178925.s008.tif]
